# Electron carriers involved in autotrophic and heterotrophic acetogenesis in the thermophilic bacterium *Thermoanaerobacter kivui*

**DOI:** 10.1007/s00792-021-01247-8

**Published:** 2021-10-14

**Authors:** Alexander Katsyv, Surbhi Jain, Mirko Basen, Volker Müller

**Affiliations:** 1 Department of Molecular Microbiology and Bioenergetics, Institute of Molecular Biosciences, Johann Wolfgang Goethe University, Frankfurt am Main, Germany; 2 Microbiology, Institute of Biological Sciences, University of Rostock, 18059 Rostock, Germany

**Keywords:** Acetogenic metabolism, Extremophile, Electron-bifurcating hydrogenase, Methylene-THF reductase

## Abstract

*Thermoanaerobacter kivui* is an acetogenic model organism that reduces CO_2_ with electrons derived from H_2_ or CO, or from organic substrates in the Wood–Ljugdahl pathway (WLP). For the calculation of ATP yields, it is necessary to know the electron carriers involved in coupling of the oxidative and reductive parts of metabolism. Analyses of key catabolic oxidoreductases in cell-free extract (CFE) or with purified enzymes revealed the physiological electron carriers involved. The glyceraldehyde-3-phosphate dehydrogenase (GA3P-DH) assayed in CFE was NAD^+^-specific, NADP^+^ was used with less than 4% and ferredoxin (Fd) was not used. The methylene-THF dehydrogenase was NADP^+^-specific, NAD^+^ or Fd were not used. A Nfn-type transhydrogenase that catalyzes reduced Fd-dependent reduction of NADP^+^ with NADH as electron donor was also identified in CFE. The electron carriers used by the potential electron-bifurcating hydrogenase (HydABC) could not be unambiguously determined in CFE for technical reasons. Therefore, the enzyme was produced homologously in *T. kivui* and purified by affinity chromatography. HydABC contained 33.9 ± 4.5 mol Fe/mol of protein and FMN; it reduced NADP^+^ but not NAD^+^. The methylene-THF reductase (MetFV) was also produced homologously in *T. kivui* and purified by affinity chromatography. MetFV contained 7.2 ± 0.4 mol Fe/mol of protein and FMN; the complex did neither use NADPH nor NADH as reductant but only reduced Fd. In sum, these analysis allowed us to propose a scheme for entire electron flow and bioenergetics in *T. kivui*.

**Supplementary Information:**

The online version contains supplementary material available at 10.1007/s00792-021-01247-8.

## Introduction

Acetogenic bacteria are a polyphyletic group and characterized by an ancient pathway for carbon dioxide fixation that is coupled to energy conservation by a chemiosmotic mechanism of ATP synthesis (Drake et al. 2008). CO_2_ is fixed by the Wood–Ljugdahl pathway (WLP), a pathway with two branches in which the methyl and the carbonyl group of acetyl-CoA are formed from one mol of CO_2_ each (Ljungdahl 1986; Wood et al. 1986). The two branches converge by forming a C–C bond, with the production of acetyl-CoA (Ragsdale and Wood 1985). In the anabolic route, acetyl-CoA is further carboxylated to pyruvate and from here, carbon flows towards the usual biosynthetic routes for the production of cell material (Fairhurst et al. 1956; Kandler 1983). In the catabolic route, acetyl-CoA is converted to acetate via acetyl-phosphate, giving rise to one mol ATP per mol of acetate (Schaupp and Ljungdahl 1974). Since one ATP is consumed in the first step of the methyl branch (Himes and Harmony 1973), the overall ATP balance by substrate level phophorylation is zero and to gain net cellular ATP, acetogens employ a chemiosmotic mechanism for ATP synthesis in addition (Schuchmann and Müller 2014). Indeed, there are two different types of respiratory enzymes present in acetogens. Both use reduced ferredoxin (Fd^2−^) as electron donor, but the electron acceptor is either H^+^ (Fd^2−^:H^+^ oxidoreductase, Ech) (Schoelmerich and Müller 2019) or NAD^+^ (Fd^2−^:NAD^+^ oxidoreductase, Rnf) (Biegel and Müller 2010). Electron transfer from reduced Fd to the electron acceptor leads to transport of ions (H^+^, Na^+^) across the cytoplasmic membrane and the built up of a transmembrane electrochemical ion potential which in turn drives the synthesis of ATP via a membrane-integral F_1_F_O_ ATP synthase (Hess et al. 2013; Schoelmerich and Müller 2019; Kuhns et al. 2020). It is important to note that acetogens have either one of these two enzymes, never both, and are, therefore, classified as Rnf- or Ech-acetogens (Schuchmann and Müller 2014). H_2_ or NADH are the “waste” products of these respirations and are re-oxidized in the WLP (Müller 2003; Katsyv and Müller 2020). Autotrophic growth of acetogens on H_2_ + CO_2_ with a free energy change of only -95 kJ/mol under standard conditions is at the thermodynamic limit; taken into account environmental H_2_ concentrations (Thauer et al. 1977; Müller 2003; Schuchmann and Müller 2014), this value goes down to around  − 20 kJ/mol, allowing for the synthesis of only ≈ 0.3 mol ATP/mol acetate (Müller 2003).

The critical number for the energetics of acetogens is the number of moles of reduced Fd available for the respiratory enzymes. This number depends on the electron carrier specificity of the enzymes of the WLP. The ultimate source of electrons is molecular hydrogen which is oxidized by electron-bifurcating hydrogenases (Schuchmann and Müller 2012; Wang et al. 2013a, b). Four moles of hydrogen are oxidized with the concomitant reduction of two moles pyridine nucleotides (NAD(P)H) and two moles reduced Fd. If more than one enzyme in the WLP requires reduced Fd as reductant, there would be a lack of electrons for the electron transport chain. Therefore, for the calculation of ATP yields, it is crucial to know the electron carrier specificity of the WLP enzymes in a given species.

The ability to utilize syngas (CO_2_, CO, H_2_) makes acetogenic bacteria to key players in the global carbon and hydrogen cycle and thus prime candidates as driving forces in a H_2_- and CO_2_-bioeconomy (Müller 2019). In the last years, tremendous progress was made in developing acetogens by metabolic engineering to convert syngas to biofuels (Daniell et al. 2012; Liew et al. 2016; Köpke and Simpson 2020) or establishing acetogens for H_2_ capture and storage in the biohydrogen economy (Schwarz et al. 2020, 2021; Müller 2019). However, the energetics of product formation from H_2_ + CO_2_ and CO are only poorly understood in most acetogens (Katsyv and Müller 2020).

The thermophilic acetogen *Thermoanaerobacter kivui* grows in mineral media with high growth rates on H_2_ + CO_2_, CO or mixtures of both (synthesis gas, syngas) (Leigh et al. 1981; Klemps et al. 1987; Hess et al. 2014; Weghoff and Müller 2016; Basen and Müller 2017). It contains the Ech complex as respiratory enzyme (Schoelmerich and Müller 2019), a H_2_-dependent CO_2_ reductase (HDCR) (Schwarz et al. 2018) as first enzyme in the carbonyl branch, a pyruvate:ferredoxin oxidoreductase (PFOR) (Katsyv et al. 2021) and a Fd^2−^-dependent CO dehydrogenase (Jain et al. 2021) (Table 1). Unforunately, the electron carrier specificity of the other redox enzymes of the WLP are unknown. Here, we have identified the electron carriers involved in these reactions, using cell-free extract (CFE) or purified proteins as experimental material. These studies allowed us to present the first nearly complete pathway of electron flow and the bioenergetics in this model acetogen.

**Table 1:** Specific activities of purified oxidoreductases involved in carbon catabolism, carbon fixation and energy metabolism of *T. kivui*

Enzyme	Substrates	Specific activity [U/mg]
Pyruvate:ferredoxin oxidoreductase (PFOR)^1^	Pyruvate + CoA + **Fd**	27.2 ± 4.1
Carbon monoxide dehydrogenase (CODH/ACS)^2^	CO + **Fd**	111.5 ± 15.4
Carbon monoxide dehydrogenase (CooS)^2^	CO + **Fd**	0.5 ± 0.03
H_2_-dependent CO_2_ reductase (HDCR)^3^	H_2_ + CO_2_ → H^+^ + **formate**	930
	Formate + H^+^ → CO_2_ + **H**_**2**_	900
Energy-converting Hydrogenase (Ech)^4^	H_2_ + **Fd**	1.2 ± 0.2
	^%^Fd^2−^ + H^+^ → **H**_**2**_	10.2 ± 3.4

Substrates or products whose reduction, oxidation, or formation were monitored are presented in bold. The activities were determined at 66 °C with purified proteins. One unit (U) equals 2 µmol of electrons transferred per min. All measurements were performed in biological replicates. Fd, ferredoxin (isolated from *C. pasteurianum*)

^%^Fd^2−^-regenerating system (Fd, TPP, CoA, pyruvate, PFOR (Katsyv et al. 2021))

^1^purified and characterized in Katsyv et al. (2021)

^2^purified and characterized in Jain et al. (2021)

^3^purified and characterized in Schwarz et al. (2018)

^4^unpublished data

## Materials and methods

### Growth of *T. kivui*

*T. kivui* (DSM 2030) was grown at 66 °C in complex medium under anoxic conditions in 1-l-bottles (Glasgerätebau Ochs, Bovenden-Lenglern, Germany) using 28 mM D-glucose as substrate (Weghoff and Müller 2016). The medium was prepared using the anaerobic techniques described previously (Hungate 1969; Bryant 1972). Growth was monitored by measuring the OD at 600 nm. Plating and cultivation on solid media were the same as described previously (Basen et al. 2018). 200 µg/ml kanamycin was used to select for recombinants.

### Preparation of CFE

All buffers used were prepared using the anaerobic techniques described previously (Hungate 1969; Bryant 1972). All purification steps were performed under strictly anaerobic conditions at room temperature in an anaerobic chamber (Coy Laboratory Products, Grass Lake, Michigan, USA) filled with 95–98% N_2_ and 2–5% H_2_. Cells of *T. kivui* were harvested and washed twice in buffer A1 (50 mM Tris/HCl, 10 mM NaCl, 20 mM MgSO_4_, 2 mM DTE, 4 µM resazurin, 20% [v/v] glycerol, pH 7.5). The cells were resuspended in 20 ml buffer A1 including 0.5 mM PMSF and 0.1 mg/ml DNAseI and passed one time through a French pressure cell (110 MPa). Cell debris was removed by centrifugation at 24,000 × *g*, 4 °C for 20 min. The supernatant contained the CFE, which was stored at 4 °C for further investigations.

### Cloning of *pMU131_His-hydABC* and *pMU131_His-metFV*

Plasmid *pMU131_His-hydABC* and *pMU131_His-metFV* were used for the expression of *hydABC* (TKV_c19580–TKV_c19600) and *metFV* (TKV_c19880–TKV_c19890) in *T. kivui*. The plasmids are based on plasmid *pMU131* (Shaw et al. 2010), that replicates in *T. kivui* and confers resistance to kanamycin (Basen et al. 2018; Katsyv et al. 2021). The inserts *hydABC* (4219 bp) and *His-metFV* (1612 bp) were amplified using the primers HydABCTK_for (3) and HydABCTK_rev (4) or His-MetFVTK_for (5) and His-MetFVTK_rev (6). The backbone *pMU131* (7192 bp) was amplified using the primers pMU131_for (1) and pMU131_rev (2), followed by the fusion of the PCR products via Gibson Assembly (Gibson Assembly Mastermix, NEB, Frankfurt/Main, Germany). A DNA sequence encoding a 10 × histidine-tag (His-tag) was introduced in pMU131_*hydABC* at the 3’-end of the gene *hydA* using corresponding primers His-HydA_for (9) and His-HydA_rev (10). *T. kivui* (DSM 2030) was transformed with the resulting plasmids *pMU131_His-hydABC* and *pMU131_His-metFV* as described previously (Basen et al. 2018). Cells were plated on agar medium containing 28 mM glucose as carbon source and 200 µg/ml kanamycin. To verify the transformation, colonies were picked and the transformed plasmids were checked using primer pairs seq1_for (7)/seq2_rev (8) binding on the *pMU131* backbone and amplifying the complete *His-hydABC* or *His-metFV* locus (Fig. S1).

### Production and purification of His-HydABC and His-MetFV

*T. kivui pMU131_His-hydABC* or *pMU131_His-metFV* cells were grown as described. All purification steps were performed under strictly anoxic conditions at room temperature in an anoxic chamber (Coy Laboratory Products, Grass Lake, Michigan, USA) filled with 95–98% N_2_ and 2–5% H_2_. Cells were harvested and washed twice in buffer A2 (50 mM Tris/HCl, 150 mM NaCl, 20 mM MgSO_4_, 10 mM imidazole, 0.5 mM DTE, 4 µM resazurin, 20% [v/v] glycerol, pH 7.5). All buffers additionally contained 10 μM FMN to avoid the loss of flavin during purification, if not otherwise specified. The cells were resuspended in 20 ml buffer A2 including 0.5 mM PMSF and 0.1 mg/ml DNAseI and passed one time through a French pressure cell (110 MPa). Cell debris was removed by centrifugation at 24,000 × *g* for 20 min. Purification of the His-tagged proteins was carried out with a nickel nitrilotriacetic acid (Ni^2+^-NTA) resin (Qiagen, Hilden, Germany) using a gravity flow column under anoxic conditions as described previously (Katsyv et al. 2021). Fractions containing His-HydABC or His-MetFV were collected, pooled, concentrated, using 50-kDa VIVASPIN tubes, and separated on a superdex 200 10/300 GL increase prepacked column (GE Healthcare Life Sciences, Little Chalfont, UK). The sample was loaded on a superdex 200 column equilibrated with buffer B (50 mM Tris/HCl, 150 mM NaCl, 20 mM MgSO_4_, 2 mM DTE, 4 µM resazurin, 20% [v/v] glycerol, pH 7.5) and eluted at a flow rate of 0.5 ml/min. HydABC or MetFV activity eluted in a single peak with a maximum at 10.2 or 11.8 ml elution volume. Fractions containing His-HydABC or His-MetFV were pooled and stored at 4 °C.

### Enzyme activity assays

All enzyme assays, unless otherwise specified, were performed in 1.8 ml anoxic cuvettes (Glasgerätebau Ochs GmbH, Bovenden-Lenglern, Germany) sealed by rubber stoppers in a N_2_ atmosphere (1 × 10^5^ Pa) at 66 °C at an overall liquid volume of 1 ml. One unit is defined as transfer of 2 µmol electrons/min. All measurements were performed in biological replicates. NAD(P)^+^/NAD(P)H was monitored spectrophotometrically at 340 nm (*ε* = 6.3 mM^−1^ cm^−1^), ferredoxin (Fd) (isolated from *Clostridium pasteurianum* (Schönheit et al. 1978)) at 430 nm (*ε* = 13.1 mM^−1^ cm^−1^) and methyl viologen (MV) or benzyl viologen (BV) at 600 nm (*ε* = 13.9 mM^−1^ cm^−1^ or *ε* = 12 mM^−1^ cm^−1^).

### Glyceraldehyde-3-phosphate dehydrogenase

Glyceraldehyde-3-phosphate dehydrogenase (GA3P-DH) activity was measured in buffer C (50 mM Tris/HCl, 10 mM NaCl, 2 mM DTE, pH 7.5), containing 200–500 μg CFE of glucose-grown cells, 5 mM arsenate and 4 mM NAD^+^/NADP^+^ or 30 μM Fd. The reaction was started by addition of 1 mM glyceraldehyde-3-phosphate (GA3P).

### Methylene-THF dehydrogenase

Methylene-THF dehydrogenase activity was measured in buffer C. The assay contained 200–500 μg CFE of glucose-grown cells, 1.5 mM formaldehyde and 0.5 mM tetrahydrofolate (THF). Formaldehyde reacts spontaneously, non-enzymatically with THF to yield methylene-THF (Kallen and Jencks 1966). The reaction was started by addition of 1 mM NAD^+^/NADP^+^ or 30 µM Fd.

### Transhydrogenase

Transhydrogenase activity was measured in buffer C. The assay contained 410 μg CFE of glucose-grown cells and 30 µM Fd. To keep the level of NADPH constant, 0.1 mM NADP^+^ were pre-reduced with 1 unit glucose-6-phosphate dehydrogenase (G6P-DH) and 20 mM glucose-6-phosphate (G6P; NADP^+^ reducing system) as reported previously (Kremp et al. 2020). The reaction was started by addition of 1 mM NAD^+^.

### Electron-bifurcating hydrogenase

The activity was measured in buffer D (50 mM EPPS, 10 mM NaCl, pH 8), containing 60–100 μg CFE of glucose-grown cells or 5–10 μg purified His-HydABC, 30 μM Fd and 5 µM FMN; the gas phase was 100% H_2_ (2 × 10^5^ Pa). The reaction was started by addition of 4 mM NADP^+^/NAD^+^. The controls omitted either Fd or NADP^+^/NAD^+^. H_2_ evolution was assayed in 7.2-ml glass vials (Glasgerätebau Ochs GmbH, Bovenden-Lenglern, Germany) with 5–20 μg His-HydABC, 30 μM Fd, 5 µM FMN and 10 mM NADPH/NADH. To reduce Fd, the assay additionally contained 5 μg PFOR (isolated from *T. kivui* (Katsyv et al. 2021)), 400 μM coenzyme A (CoA) and 100 μM thiamine pyrophosphate (TPP). The reaction was started by addition of pyruvate at a final concentration of 10 mM. H_2_ was measured via gas chromatography as discribed previously (Schoelmerich and Müller 2019). The controls omitted either Fd or NADPH/NADH. To determine H_2_:MV oxidoreductase activity, the assay contained 0.5–20 μg purified His-HydABC or proteins from different purifications steps. The gas phase was 100% H_2_ (2 × 10^5^ Pa). The reaction was started by addition of 10 mM MV. The effect of CO on the H_2_:MV oxidoreductase activity of His-HydABC was measured with CO concentrations (in the aqueous phase) ranged between 0–187 µM, respectively. For *K*_m_ determination, the H_2_, NADP^+^ and Fd concentrations ranged between 0–325 µM, 0–4 mM and 0–200 µM, respectively. For the determination of the pH and temperature profile, the assay containing His-HydABC was preincubated for 10 min at the pH or temperature indicated. The pH optima were determined in buffer E containing 50 mM MES, 50 mM CHES, 50 mM CAPS, 50 mM Bis–Tris, 50 mM Tris, 10 mM NaCl, 4 mM DTE, 4 μM resazurin at pH 5–10, as specified in the experiments.

### Methylene-THF reductase

Methylene-THF reductase (MTHFR) activity was measured in buffer F (50 mM KP_i_, 20 mM ascorbate, pH 7). The MV^2−^:methylene-THF oxidoreductase assay contained 0.5 mM THF, 1.5 mM formaldehyde and 10 mM MV. To reduce MV, 5 mM sodium dithionite was added. The reaction was started by addition of 200 μg CFE, 2–5 μg His-MetFV or 10 μg proteins from different purifications steps. To determine Fd^2−^:methylene-THF oxidoreductase activity the assay contained 30 μg His-MetFV, 0.5 mM THF and 1.5 mM formaldehyde. To reduce Fd, the assay additionally contained 5 μg PFOR, 400 μM CoA and 100 μM TPP. The reaction was started by addition of 10 mM pyruvate. In the controls, either Fd or methylene-THF was omitted. Methyl-THF was determined via HPLC as described recently (Dietrich et al. 2021). To determine methyl-THF:NAD^+^/NADP^+^/Fd/BV oxidoreductase the assay contained 400 μg CFE or 15–30 μg His-MetFV and 4 mM NAD^+^/NADP^+^, 30 μM Fd or 10 mM BV. The reaction was started by addition of 1 mM methyl-THF. To determine NADH/NADPH:methylene-THF oxidoreductase, the assay contained 400 μg CFE or 20 μg His-MetFV, 1.5 mM formaldehyde and 0.5 mM THF. The reaction was started by addition of 0.5 mM NADH/NADPH. To determine the Fd-dependent NADH/NADPH:methylene-THF oxidoreductase activity the assay contained 20 μg His-MetFV or 400 μg CFE, 0.5 mM THF, 0.5 mM NADH/NADPH and 30 μM Fd. The reaction was started by addition of 1.5 mM formaldehyde. To determine the NADH/NADPH:BV oxidoreductase activity, the assay contained 30 μg His-MetFV and 0.5 mM NADH/NADPH. The reaction was started by addition of 10 mM BV. For the determination of the pH and temperature profile, the assay containing His-MetFV was preincubated for 10 min at the pH or temperature indicated. The pH optima were determined in buffer E containing 50 mM MES, 50 mM CHES, 50 mM CAPS, 50 mM Bis–Tris, 50 mM Tris, 10 mM NaCl, 4 mM DTE, 4 μM resazurin at pH 5–10, as specified in the experiments.

### Analytical methods

The concentration of proteins was measured according to Bradford (1976). Proteins were separated in 12% polyacrylamide gels and stained with Coomassie brilliant blue G250. The iron content of the purified enzymes was determined by colorimetric methods (Fish 1988), flavin was analyzed by thin-layer chromatography (TLC) (Bertsch et al. 2013). The molecular mass of the purified His-HydABC and His-MetFV was determined using a calibrated superdex 200 column and defined size standards (ovalbumin: 43 kDa; albumin: 158 kDa; catalase: 232 kDa; ferritin: 440 kDa).

## Results and discussion

### Electron carrier specificity of catabolic oxidoreductases examined in CFE of glucose-grown cells

To analyze the electron carrier specificity of the glycolytic enzyme GA3P-DH, oxidation of GA3P by CFE of glucose-grown cells was analyzed. The CFE of *T. kivui* catalyzed GA3P oxidation coupled to NAD^+^ reduction with an activity of 0.85 ± 0.15 U/mg (Fig. S2A). In contrast, NADP^+^ was reduced with very low rates (< 0.03 U/mg) (Fig. S2B) and Fd did not serve as electron acceptor (Fig. S2C), demonstrating that NAD^+^ is the cofactor used by the GA3P-DH.

The CFE also catalyzed the reduction of NADP^+^ with methylene-THF as electron donor (24.4 ± 1.2 U/mg) (Fig. S3A). NAD^+^ (Fig. S3B) or Fd (Fig. S3C) were not reduced, demonstrating a NADPH-dependent methylene-THF dehydrogenase. The MTHFR of *T. kivui* is of the MetFV-type (*metF*, TKV_c19880 and *metV*, TKV_c19890) (Hess et al. 2014; Öppinger et al. 2021). Methylene-THF was not reduced with NADPH or NADH as reductant in CFE. The reverse reaction, oxidation of methyl-THF was not coupled to NAD^+^, NADP^+^ or Fd reduction, but only to BV reduction (2.4 ± 0.7 U/mg). Electron bifurcation (Fd-dependent NADH/NADPH:methylene-THF oxidoreductase) was also not observed.

The genome of *T. kivui* codes for an electron-bifurcating, Fd-dependent transhydrogenase, NfnAB (TKV_c22270–TKV_c22280) to transfer electrons between NAD^+^ and NADP^+^ (Hess et al. 2014). CFE of *T. kivui* catalyzed the reduction of NAD^+^ with NADPH only in the presence of Fd. The NAD^+^-dependent NADPH:Fd oxidoreductase activity was 0.04 ± 0.01 U/mg (Fig. S4A). When Fd (Fig. S4B) or NADPH (Fig. S4C) was omitted, no NAD^+^ reduction was observed, indicating a functional NfnAB complex. All oxidoreductase activities measured in CFEs of glucose-grown *T. kivui* cells are summarized in Table 2.

**Table 2:** Oxidoreductase activities in CFE of glucose-grown *T. kivui* strain

Enzyme	Substrates	Specific activity [U/mg]
Methylene-THF dehydrogenase	Methylene-THF + **NADP**^**+**^	24.4 ± 1.2
	Methylene-THF + **NAD**^**+**^	< 0.005
	Methylene-THF + **Fd**	< 0.001
NAD^+^-dependent NADPH:Fd oxidoreductase (Nfn)	^#^NADPH + NAD^+^ + **Fd**	0.04 ± 0.01
	^#^NADPH + **NAD**^**+**^	< 0.001
	NADH + **NADP**^**+**^	< 0.002
	^#^NADPH + **Fd**	< 0.001
Glyceraldehyde-3-phosphate dehydrogenase (GA3P-DH)	GA3P + **NADP**^**+**^	< 0.03
	GA3P + **NAD**^**+**^	0.85 ± 0.15
	GA3P + **Fd**	< 0.001

Substrates whose reduction, oxidation, or formation were monitored are presented in bold. The activities were determined at 66 °C. One unit (U) equals 2 µmol of electrons transferred per min. All measurements were performed in biological replicates. For more details see Materials and methods

Fd, ferredoxin (isolated from *C. pasteurianum*); THF, tetrahydrofolate

^#^NADPH-regenerating system (NADP^+^, G6P, G6P-DH (Kremp et al. 2020)

### Identification of a NADP^+^-dependent H_2_:Fd oxidoreductase activity in *T. kivui* and purification of the electron-bifurcating hydrogenase

**Fig. 1:**
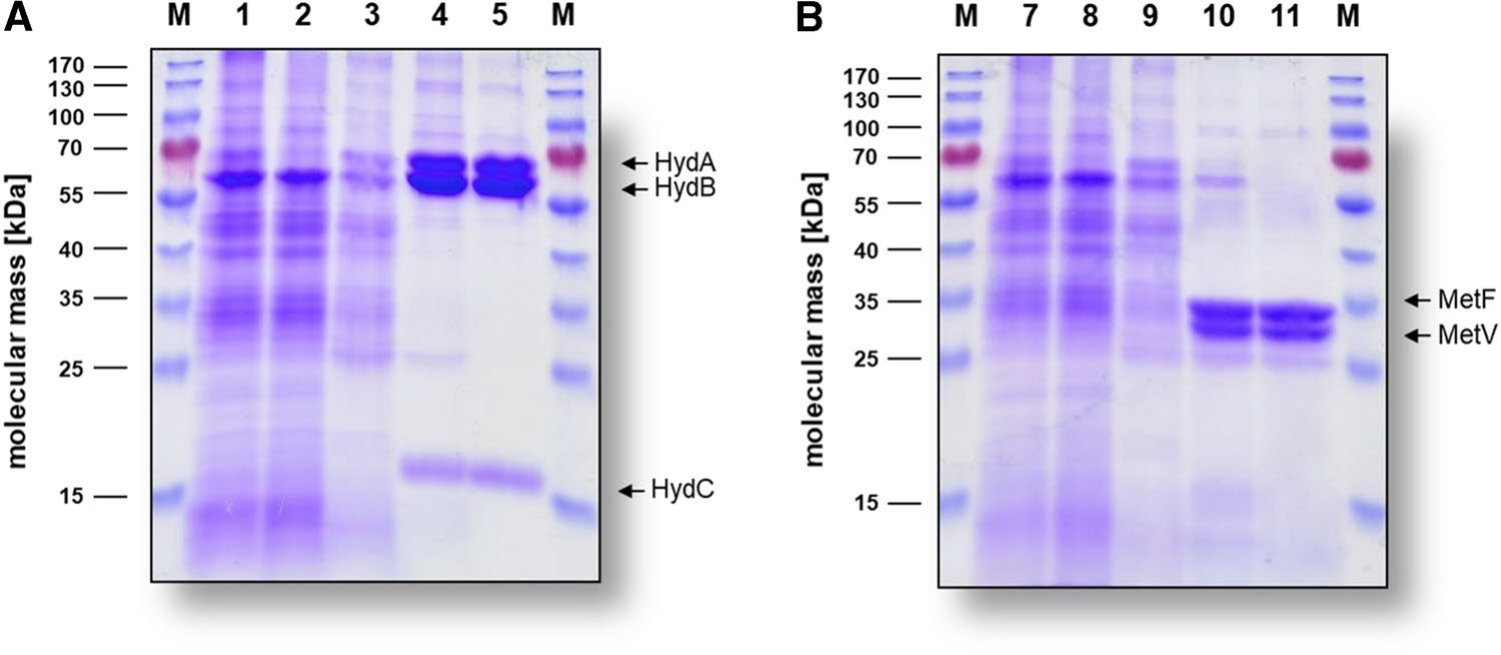
SDS-PAGE monitoring the purification process of His-HydABC and His-MetFV. Samples of the different purification steps of His-HydABC (**A**) and His-MetFV (**B**) were separated by SDS-PAGE (12%) and proteins were stained with Coomassie Brilliant Blue G250. 10 µg of protein was applied to each lane. M, prestained page ruler; lane 1 and 7, CFE; lane 2 and 8, flow through; lane 3 and 9, wash fraction; lane 4 and 10, pooled Ni^2+^-NTA elution fractions; lane 5 and 11, pooled size exclusion fractions

The genome of *T. kivui* encodes an electron-bifurcating hydrogenase HydABC (*hydC*, *hydB*, *hydA*; TKV_c19580–c19600 cluster) (Hess et al. 2014), which is very similar to the NAD^+^-dependent H_2_:Fd hydrogenase HydABC of *Acetobacterium woodii* (HydC: 44%, HydB: 58%, HydA: 48%; Awo_c27010–c26970 cluster) (Schuchmann and Müller 2012) and *Moorella thermoacetica* (HydC: 49%, HydB: 57%, HydA: 56%; MOTHA_18110–18090 cluster) (Wang et al. 2013a, b) or to the NADP^+^-dependent H_2_:Fd hydrogenase of *Clostridium autoethanogenum* (HydC: 47%, HydB: 59%, HydA: 54%; CAETHG_3571–3569 cluster) (Wang et al. 2013a, b) and *Clostridium ljungdahlii* (HydC: 45%, HydB: 55%, HydA: 53%; CLJU_c14720–14,700 cluster) (Nagarajan et al. 2013; Wang et al. 2013a, b). When testing for NAD^+^-dependent H_2_:Fd oxidoreductase activity in CFE of *T. kivui*, Fd and NAD^+^ reduction with H_2_ as reductant was only weak (0.02 ± 0.01 U/mg). Suprisingly, NADP^+^ reduction with H_2_ as electron donor in the presence of Fd was stronger (0.05 ± 0.02 U/mg). However, Fd reduction was also measurable with H_2_ as electron donor alone, indicating interference with other enzymes in the CFE. To determine the cofactor specificity of the isolated HydABC hydrogenase of *T. kivui*, we took advantage of a plasmid-based production system in *T. kivui* (Katsyv et al. 2021) to produce and purify the enzyme. Therefore, we cloned *hydABC* together with a DNA sequence coding for a 10 × His-tag into *pMU131* (Fig. S5A-D). Naturally competent cells of *T. kivui* were transformed with the plasmid (Fig. S1A, B), grown on glucose and CFE was prepared. The complex was purified via a genetically engineered His-tag at HydA to apparent homogeneity by Ni^2+^-NTA-sepharose followed by size exclusion chromatography on Superdex 200. Using this procedure, the enzyme was purified 424-fold to apparent homogeneity with an average specific H_2_:MV oxidoreductase activity of 7596.0 ± 2370.3 U/mg and a yield of 3.2 mg (per 2 g wet cells) (Table 3). Analyses of the purified His-HydABC separated on a 12% SDS–polyacrylamide gel revealed three proteins with apparent molecular masses of ≈ 65, 70 and ≈ 18 kDa (Fig. 1A). These molecular masses correspond well with the expected sizes for HydA (TKV_c19600, 64 kDa), HydB (TKV_c19590, 68 kDa) and HydC (TKV_c19580, 18 kDa) of *T. kivui*. Analytical size exclusion chromatography revealed a molecular mass of 348 kDa for the purified complex, which is consistent with HydABC being a dimer. We measured 33.9 ± 4.5 mol of iron/mol of protein, which matches the prediction that HydABC contains seven [4Fe–4S] cluster, three [2Fe–2S] and one [FeFe] cluster. All current characterized bifurcating enzymes possess either a quinone or flavin group with special redox properties (Müller et al. 2018). From bioinformatic analyses, HydABC should contain one flavin, but the nature of the flavin remained to be established. After precipitation of the purified complex and subsequent separation of the flavin-containing supernatant by thin-layer chromatography (TLC), no FAD, but FMN was detected (Fig. S6A).

**Table 3:** Purification of His-HydABC from *T. kivui*

Purification step	Protein [mg]	HydABC activity* [U/mg]	Purification [-fold]	Yield [%]
CFE	378.4	17.9	1	100
Ni^2+^-NTA	4.2	6681.2	373.3	1.1
Superdex 200	3.2	7596.0	424.4	0.8

^*^HydABC activity was measured with H_2_ as electron donor and MV as electron acceptor

**Fig. 2:**
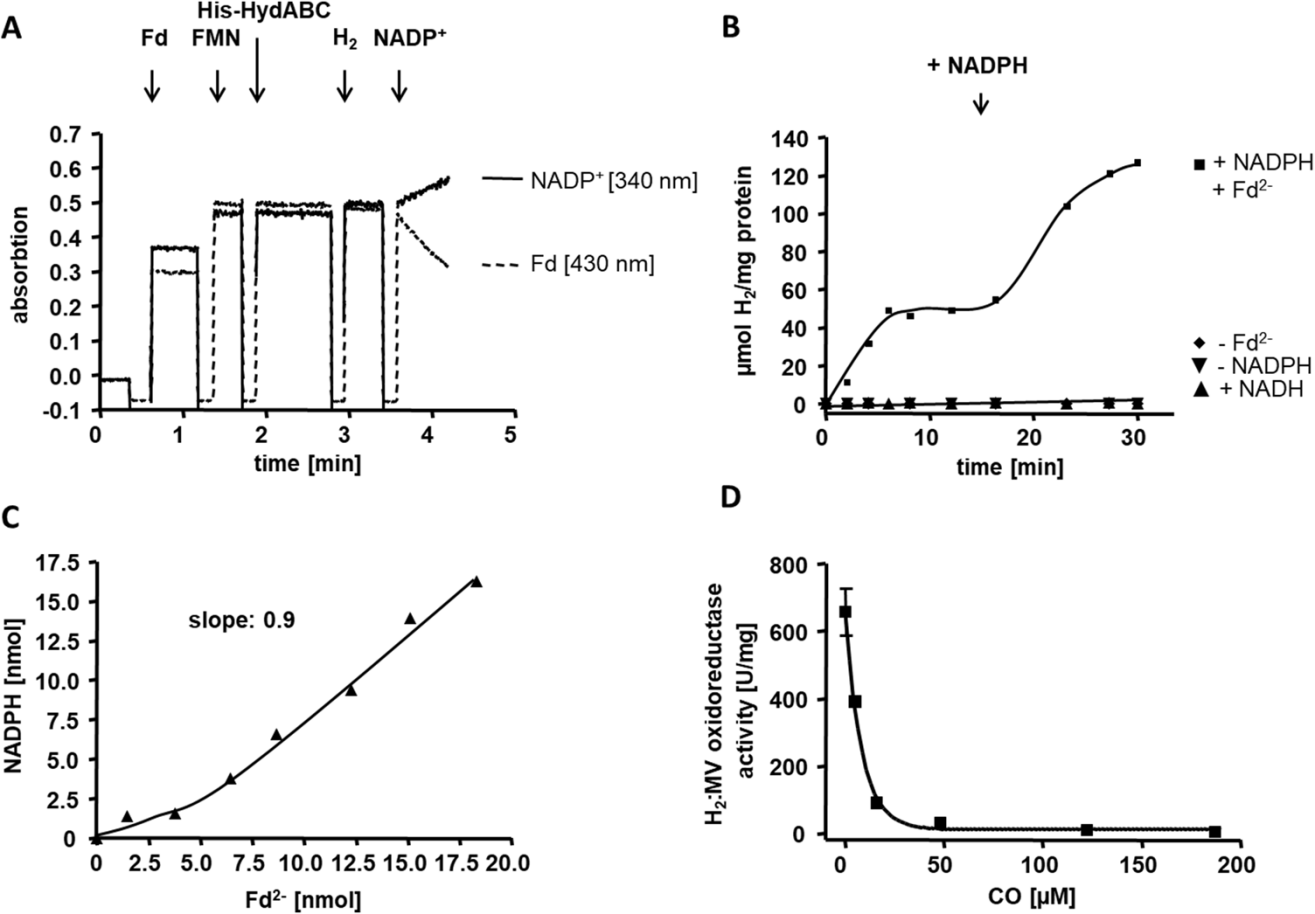
Electron-bifurcating activity of the purified His-HydABC complex. Simultaneous hydrogen-dependent reduction of Fd and NADP^+^ (**A**) was measured in 1.8-ml anoxic cuvettes containing an overall liquid volume of 1 ml under a 100% H_2_ atmosphere (2 × 10^5^ Pa) at 66 °C. The assay contained 10 μg His-HydABC, 5 µM FMN and 30 μM Fd served as electron acceptor in buffer D (50 mM EPPS, 10 mM NaCl, pH 8). The reaction was started by addition of 4 mM NADP^+^. Reduction of NADP^+^ (continuous line, monitored at 340 nm) and reduction of Fd (dashed line, monitored at 430 nm) were monitored simultaneously. Hydrogen evolution from NADPH and Fd^2−^ (**B**) was measured via gas chromatography. The assay contained buffer D,  20 μg His-HydABC, 30 μM Fd, 5 µM FMN and 10 mM NADPH (■). To reduce Fd (isolated from *C. pasteurianum*) the assay additionally contained 5 μg PFOR (isolated from *T. kivui*), 400 μM CoA and 100 μM TPP. The reaction was started with 10 mM pyruvate. After 18 min additional 10 mM NADPH was added as indicated. In the controls either Fd (◆) or NADPH (▼) was omitted or NADPH was replaced by NADH (▲). Stoichiometry of Fd and NADP^+^ reduction was calculated from the absorbance difference in (A). To calculate the ratio the amount of reduced NADP^+^ is plotted against the amount of reduced Fd (**C**). CO inhibition experiments were performed with different CO concentrations in the assay (**D**). The assay contained 5–10 μg His-HydABC, H_2_ in the atmosphere (1 × 10^5^ Pa) and 10 mM MV in buffer D. The specific H_2_:MV oxidoreductase activity was ploted against the CO concentration

The purified complex catalyzed electron bifurcation from H_2_ to Fd and NADP^+^ with an activity of 20.8 ± 2.5 U/mg (Fig. 2A). When NADP^+^ was replaced with NAD^+^ no activity was measurable (< 0.005 U/mg), which is contrary to previous assumptions (Basen and Müller 2017; Jain et al. 2020; Hess et al. 2014; Moon et al. 2020; Weghoff and Müller 2016). Furthermore, H_2_:Fd oxidoreductase activity was not detectable in the absence of NADP^+^, demonstrating that reduction of one electron acceptor (NADP^+^) was strictly dependent on the presence of the other electron acceptor (Fd). When both electron acceptors were present, they were reduced simultaneously with a 1:1 stoichiometry (slope: 0.9, Fig. 2C). FMN stimulated the HydABC activity, as described also for the purified HydABC complex of *A. woodii* (Schuchmann and Müller 2012). Purified His-HydABC exhibited a NADP^+^-dependent H_2_:Fd oxidoreductase activity of 15.6 ± 1.5 U/mg when FMN was omitted from the enzyme assay as opposed to 20.8 ± 2.5 U/mg with 5 µM FMN in the assay. It is important to note here, that FMN was also ommitted from buffer B during the last purification step to ensure no external FMN in the assay. The purified complex also catalyzed the reverse reaction, reduction of H^+^ with NADPH and reduced Fd as electron donors with an average specific activity of 9.3 ± 2.0 U/mg (Fig. 2B). All activities of His-HydABC are summarized in Table 4. Because *T. kivui* is able to convert CO as sole carbon and energy source, we analyzed whether the bifurcating hydrogenase is inhibited by CO. Therefore, we tested H_2_:MV oxidoreductase activity in the presence of different CO concentrations (Fig. 2D). Indeed, the enzyme was inhibited by CO and 50% inhibition was observed with 5.7 ± 1.2 µM CO (soluble in water), indicating a strong inhibition by low CO concentrations in vitro.

**Table 4:** Reactions catalyzed by purified His-HydABC and His-MetFV from *T. kivui*

Enzyme	Substrates	Specific activity [U/mg]
Bifurcating hydrogenase (HydABC)	H_2_ + NADP^+^ + **Fd**	20.8 ± 2.5
	H_2_ + NAD^+^ + **Fd**	< 0.01
	H_2_ + **Fd**	< 0.003
	H_2_ + **NADP**^**+**^	< 0.005
	H_2_ + **NAD**^**+**^	< 0.003
	H_2_ + **MV**	7596.0 ± 2370.3
	NADPH + ^%^Fd^2−^ + H^+^ ** → H**_**2**_	9.3 ± 2.0
	NADH + ^%^Fd^2−^ + H^+^ ** → H**_**2**_	< 0.002
Methylene-THF reductase (MetFV)	Methylene-THF + **NADPH**	< 0.002
	Methylene-THF + **NADH**	< 0.001
	Methylene-THF + **NADH + Fd**	< 0.003
	Methylene-THF + **NADPH + Fd**	< 0.002
	Methylene-THF + **MV**^**2−**^	891.8 ± 96.5
	Methylene-THF + ^%^Fd^2−^ → **methyl-THF**	0.4 ± 0.04
	Methyl-THF + **BV**	31.2 ± 5.7
	NADH + **BV**	0.2 ± 0.04
	NADPH + **BV**	0.2 ± 0.04

Substrates or products whose reduction, oxidation, or formation were monitored are presented in bold. The activities were determined at 66 °C. One unit (U) equals 2 µmol of electrons transferred per min. All measurements were performed in biological replicates. For more details see Materials and methods

Fd, ferredoxin (isolated from *C. pasteurianum*); MV, methyl viologen; BV, benzyl viologen; THF, tetrahydrofolate

^%^Fd^2−^-regenerating system (Fd, TPP, CoA, pyruvate, PFOR (Katsyv et al. 2021))

We assessed key biochemical properties of the purified His-HydABC including the temperature and pH profile as well as substrate affinities. To ensure an ideal reflection of the physiological conditions, we exclusively used the NADP^+^-dependent H_2_:Fd oxidoreductase assay. His-HydABC was active at temperatures ranging from 22 to 85 °C with a maximal activity of 26.8 ± 2.9 U/mg at the optimal growth temperature of *T. kivui* (66 °C) (Fig. S7A). At mesophilic conditions, His-HydABC activity was decreased by 86% at 22 °C and by 58% at 40 °C. At 85 °C, the activity decreased by 50%. The pH range was relatively narrow with only 22% and 55% activity at pH 6 and 10 and an optimal activity of 19.3 ± 2.4 U/mg at pH 8 (Fig. S7B). At pH 6, the activity was decreased by 78% and completely abolished at pH 5.

Next, we assessed the K_m_ values for all reaction partners of His-HydABC. The dependence of the NADP^+^-dependent H_2_:Fd oxidoreductase reaction on H_2_, Fd, NADP^+^ and FMN was hyperbolic with saturation at 160 µM H_2_ (soluble in water), 30 µM Fd, 0.2 mM NADP^+^ and 2 µM FMN, respectively (Fig. S8). The Km values of His-HydABC for H_2_, Fd, NADP^+^ and FMN were 27.5 ± 4.5 µM, 13.6 ± 3.3 µM, 48.8 ± 10.6 µM and 0.3 ± 0.8 µM, respectively (Fig. S8). Unsurprisingly, the absence of H_2_, Fd or NADP^+^ led to a complete loss of activity.

### Purification of the MTHFR

One of the biggest current uncertainties in the bioenergetics of acetogenic microorganisms is the energetics of the MTHFR reaction. The redox potential of the methylene-/methyl-THF couple of − 200 mV (Wohlfarth et al. 1990) does not allow a direct reduction of NAD^+^ or NADP^+^. One solution to this problem is electron bifurcation: Cooxidation of reduced Fd by a possible electron-bifurcating MTHFR could solve the problem. This potential complex would then transfer electrons from NADH/NADPH to methylene-THF and an additional, unidentified electron acceptor in the reductive path (Mock et al. 2014). Whether or not the MTHFR uses electron bifurcation and if so, what the second electron acceptor might be, still has to be elucidated. The picture is complicated by the fact that four different types of MTHFR´s with different subunit compositions are found in acetogens. Some definitely do not bifurcate, others maybe (Öppinger et al. 2021). Therefore, we decided to purify and charcterize the MTHFR of *T. kivui*.

In most acetogens, the core subunits of MTHFR are MetF and MetV (Clark and Ljungdahl 1984; Mock et al. 2014; Bertsch et al. 2015; Jeong et al. 2015; Visser et al. 2016; Öppinger et al. 2021). First inspection of genomic data of *T. kivui* had indicated the presence of *metF* (TKV_c19880) and *metV* (TKV_c19890) (Hess et al. 2014). No *hdr* and *mvhD* genes, as described for *M. thermoacetica* (Mock et al. 2014) or *Sporomusa ovata* (Visser et al. 2016) or a *rnfC2*-like gene, which was discribed for the NADH-dependent MTHFR of *A. woodii* (Bertsch et al. 2015), are present in the genome of *T. kivui* (Hess et al. 2014). To purify MetFV, *metFV* was cloned together with a DNA sequence coding for a 10 × His-tag into *pMU131* (Fig. S5E-H) and transformed into *T. kivui* (Fig. S1C, D). The His-tagged MetFV was purified from the CFE of glucose-grown cells to apparent homogeneity by Ni^2+^-NTA-sepharose followed by size exclusion chromatography on Superdex 200. Using this procedure, the enzyme was purified 282-fold to apparent homogeneity with an average specific MV^2−^:methylene-THF oxidoreductase activity of 593.6 ± 161.5 U/mg and a yield of 0.4 mg (per 2 g wet cells) (Table 5). Analyses of the purified His-MetFV separated on a 12% SDS–polyacrylamide gel revealed two proteins with apparent molecular masses of ≈ 33 and ≈ 28 kDa (Fig. 1B). These molecular masses correspond well with the expected sizes for MetV (23.5 kDa) and MetF (31.5 kDa) of *T. kivui*. Analytical size exclusion chromatography revealed a molecular mass of 174 kDa for the purified complex, which is consistent with three MetFV heterodimers. We measured 7.2 ± 0.4 mol of iron/mol of protein, which matches the prediction that MetFV contains two [4Fe–4S] cluster. From bioinformatic analyses, MetFV should contain one flavin, but the nature of the flavin remained to be established. After precipitation of the purified complex and subsequent separation of the flavin-containing supernatant by thin-layer chromatography (TLC), FMN could be detected (Fig. S6B).

**Table 5:** Purification of His-MetFV from *T. kivui*

Purification step	Protein [mg]	MetFV activity^+^ [U/mg]	Purification [-fold]	Yield [%]
CFE	356.2	2.1	1	100
Ni^2+^-NTA	1.7	532.5	253.6	0.5
Superdex 200	0.4	593.6	282.6	0.2

^+^MetFV activity was measured with reduced MV as electron donor and methylene-THF as electron acceptor

Next, we assessed key biochemical properties of the purified His-MetFV including the temperature and pH profile. Therefore, we used the MV^2−^:methylene-THF oxidoreductase assay. His-MetFV was active at temperatures ranging from 22 to 85 °C with a maximal activity of 481.2 ± 34.9 U/mg at the optimal growth temperature of *T. kivui* (66 °C) (Fig. S9A). At mesophilic conditions, His-MetFV activity was decreased by 88% at 22 °C and by 56% at 40 °C. At 75 and 85 °C, the activity decreased only by 9%. The pH range was relatively narrow with 89% and 67% activity at pH 6 and 8 and an optimal activity of 258.7 ± 43.4 U/mg at pH 7 (Fig. S9B). In contrast, the activity at pH 5 and 9 was almost abolished.

**Fig. 3:**
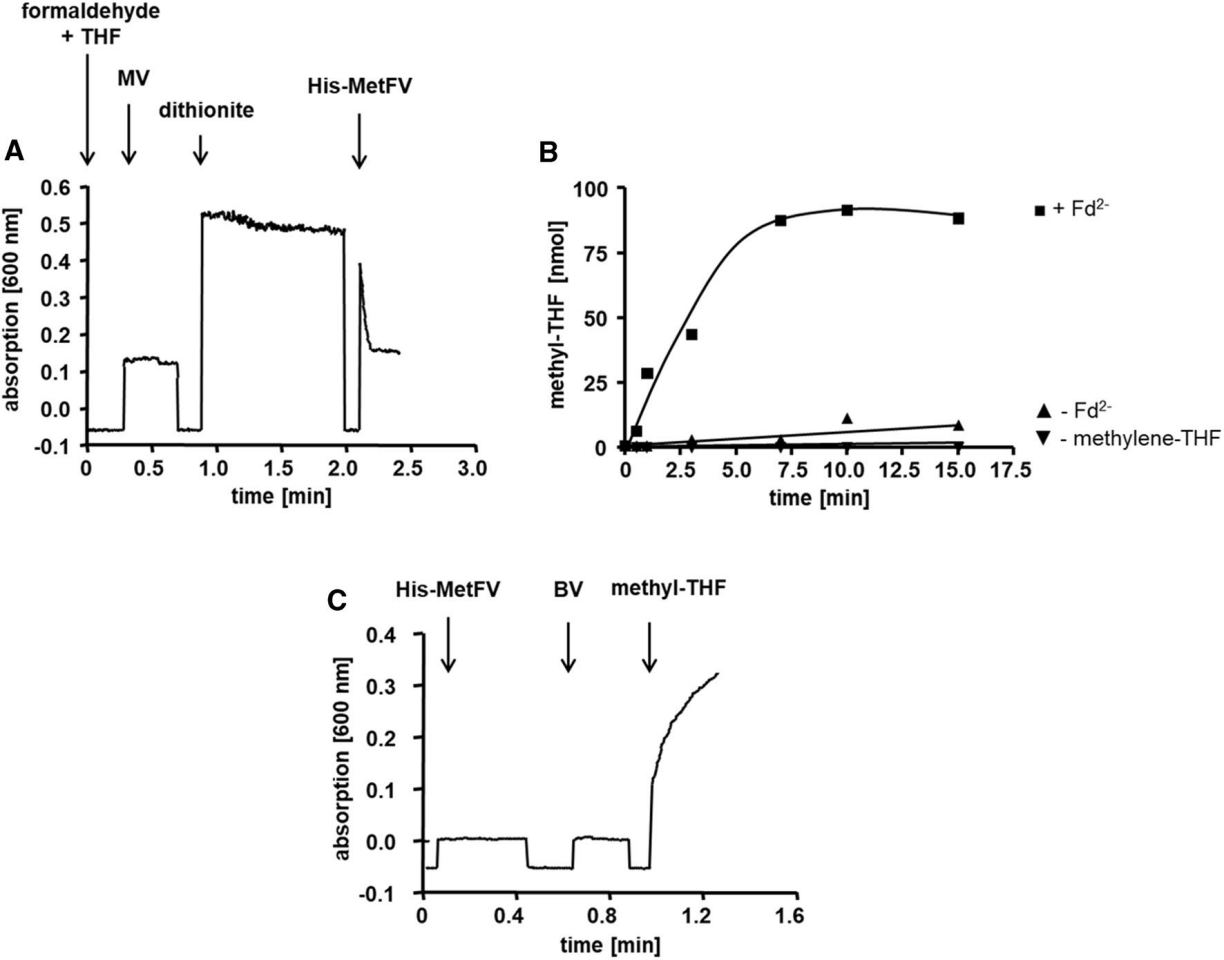
Oxidoreductase activity of the purified His-MetFV. MV^2−^:methylene-THF (**A**), Fd^2−^:methylene-THF (**B**) and methyl-THF:BV oxidoreductase activity (**C**) was measured in 1.8-ml anoxic cuvettes containing an overall liquid volume of 1 ml under a 100% N_2_ atmosphere (1 × 10^5^ Pa) at 66 °C. MV^2−^:methylene-THF oxidoreductase assay contained 0.5 mM THF, 1.5 mM formaldehyde, 10 mM MV and 5 mM sodium dithionite in buffer F (50 mM KP_i_, 20 mM ascorbate, pH 7). The reaction was started by addition of 5 μg His-MetFV. Fd^2−^:methylene-THF oxidoreductase activity assay contained 30 μg His-MetFV, 30 μM Fd, 0.5 mM THF and 1.5 mM formaldehyde in buffer F (**C**). To reduce Fd (isolated from *C. pasteurianum*) the assay additionly contained 5 μg PFOR (isolated from *T. kivui*), 400 μM CoA and 100 μM TPP. The reaction was started by addition of 10 mM pyruvate (■). In the controls either Fd (▲) or methylene-THF (▼) was omitted. After 0.5, 1, 3, 7, 10 and 15 min the amount of produced methyl-THF was meassured via HPLC, respectively (Dietrich et al. 2021). The methyl-THF:BV oxidoreductase activity assay contained 30 μg purified His-MetFV and 1 mM methyl-THF in buffer F. The reaction was started by addition of 10 mM BV. Oxidation of MV or reduction of BV was measured at 600 nm, respectively

The purified MetFV complex catalyzed the reduction of methylene-THF with reduced MV at a specific activity of 593.6 ± 161.5 U/mg (Fig. 3A). The complex did not catalyze the oxidation of NADH or NADPH in the presence of methylene-THF (Fig. S10A, B). NADH/NADPH:BV oxidoreductase activity was weak with only 0.2 ± 0.04 U/mg (Fig. S11). We also tested whether Fd (isolated from *C. pasteurianum*) was reduced by NADH or NADPH in the presence of methylene-THF, which was not the case (Fig. S12A, B). Besides reduced MV, we could identify reduced Fd as possible electron donor for the reduction of methylene-THF to methyl-THF in vitro. In the Fd^2−^:methylene-THF oxidoreductase assay, Fd was kept in a reduced state by the PFOR, isolated from *T. kivui* (Katsyv et al. 2021). From the production of methyl-THF over time, a specific activity of 0.4 ± 0.04 U/mg was calculated (Fig. 3B). The reverse reaction, oxidation of methyl-THF was not coupled to NAD^+^, NADP^+^ or Fd reduction (Fig. S13–C), but only to BV reduction (115.0 ± 42.2 U/mg) (Fig. 3C). Therefore, like in any other purified MetFV-type MTHFRs, the physiological electron carrier involved in methylene-THF reduction remains enigmatic. All activities of His-MetFV observed in this work are summarized in Table 4.

**Fig. 4:**
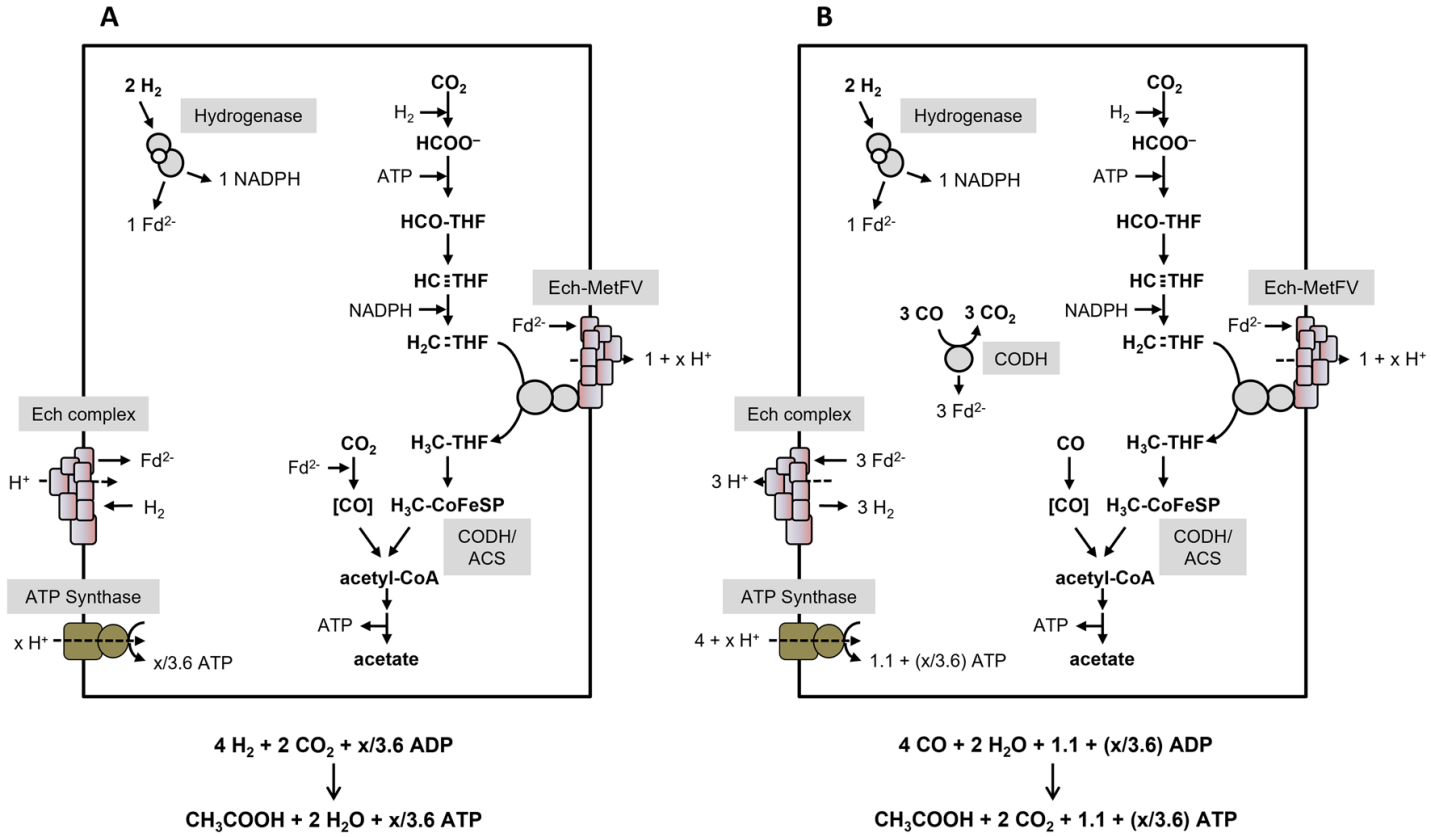
Bioenergetics of acetate formation from H_2_ + CO_2_ or CO in *T. kivui*. Acetate is formed from H_2_ + CO_2_ (**A**) or CO (**B**). The reducing equivalents in the WLP are provided by an H_2_-oxidizing, electron-bifurcating hydrogenase which reduces Fd and NADP^+^. We assume methylene-THF reduction by a membrane-coupled Ech-MetFV complex, which pumps 1 + x H^+^/2 e^−^ across the membrane (Öppinger et al. 2021). Please note that *T. kivui* has two gene clusters encoding Ech complexes with slightly different subunit compositions. Ech-MetFV complex (**A**) and Ech complex (**B**) builds up a H^+^ gradient across the cytoplasmatic membrane. This gradient drives ATP synthesis via the H^+^-dependent ATP synthase. In total, x/3.6 ATP or 1.1 + (x/3.6) ATP can be synthesized per acetate produced from H_2_ + CO_2_ or CO. Adapted from Öppinger et al. (2021). Assumed stoichiometries: H^+^/ATP = 3.6 (ATP synthase), 1 H^+^/2 e^−^ (Ech) and 1 + x H^+^/2 e^−^ (Ech-MetVF). ATP gain might be enhanced by x/3.6 ATP. CODH/ACS, CO dehydrogenase/acetyl coenzyme A synthase; THF, tetrahydrofolic acid; Ech, Energy-converting Hydrogenase

**Fig. 5:**
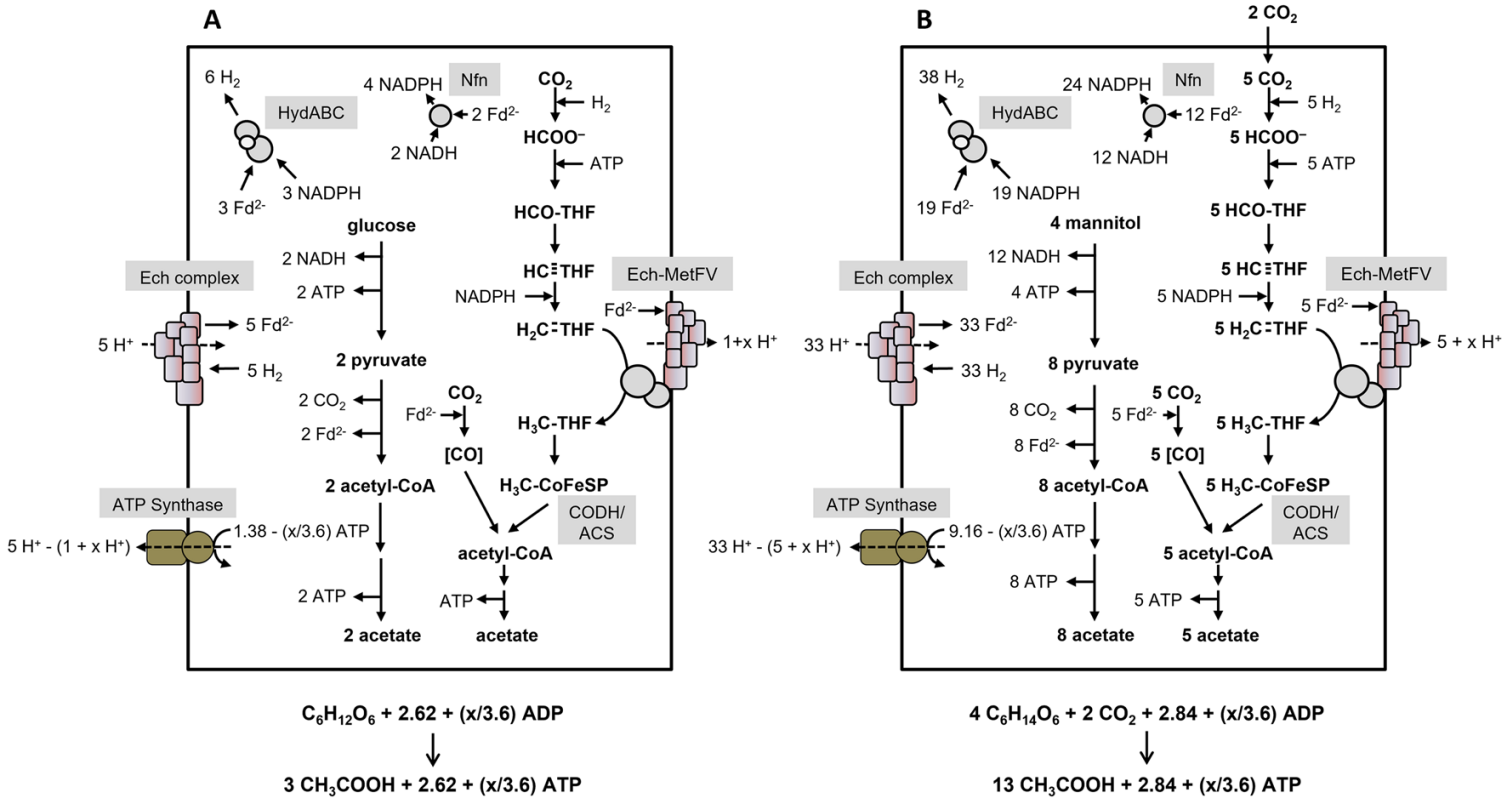
Bioenergetics of acetate formation from glucose or mannitol in *T. kivui*. Glucose (**A**) or mannitol (**B**) are converted via glycolysis to acetate. Produced and supplied CO_2_ and reducing equivalents are further utilized via the WLP, giving rise for an additional acetate. Fd^2−^ is oxidized by the bifurcating hydrogenase and the Nfn complex to produce H_2_ and to reduce NADP^+^, respectively. Hydrogen is further oxidized to reduce CO_2_ by the HDCR. Fd is reduced with H_2_ as electron donor by the Ech complex, fueled by the H^+^ gradient formed by the H^+^-dependent ATPase. We assume methylene-THF reduction by a membrane-coupled Ech-MetFV complex, which pumps 1 + x H^+^/2 e^−^ across the membrane (Öppinger et al. 2021). Please note that *T. kivui* has two gene clusters encoding Ech complexes with slightly different subunit compositions. In total, 0.87 + (x/10.8) ATP or 0.22 + (x/46.8) ATP can be synthesized per acetate produced from glucose or mannitol. Assumed stoichiometries: H^+^/ATP = 3.6 (ATP synthase), 1 H^+^/2 e^−^ (Ech) and 1 + x H^+^/2 e^−^ (Ech-MetVF). ATP gain might be enhanced by x/3.6 ATP.  CODH/ACS, CO dehydrogenase/acetyl coenzyme A synthase; THF, tetrahydrofolic acid; Nfn, transhydrogenase; Ech, Energy-converting Hydrogenase; HDCR, hydrogen-dependent CO_2_ reductase

Recently, the MetFV-type MTHFR of *C. ljungdahlii* (Öppinger et al. 2021) and *Eubacterium callanderi* (Dietrich et al. 2021), was shown to exhibit a Fd^2−^:methylene-THF oxidoreductase activity in vitro as well. Fd-dependent methylene-THF reduction by MetFV would lead to a negative ATP yield during acetogenesis from H_2_ + CO_2_, therefore, reduced Fd cannot be the physiological electron donor for *C. ljungdahlii* and *Eubacterium callanderi*. This is also true for the bioenergetics of *T. kivui*. Therefore, it was hypothesized that methylene-THF reduction is directly coupled to a respiratory complex (Öppinger et al. 2021). In case of *T. kivui*, a membrane-coupled Ech-MetFV complex was assumed (Öppinger et al. 2021). Please note that *T. kivui* has two gene clusters encoding Ech complexes with slightly different subunit compositions (Hess et al. 2014). The exact thermodynamics for the Ech complex of *T. kivui* remained to be established, nevertheless it is plausible to assume a H^+^/2 e^−^ stoichiometry for the Fd^2−^:H^+^ oxidoreductase of 1 to calculate ATP yields during growth. However, the free energy change is drastically increased, if methylene-THF serves as final electron acceptor (Δ*G*_0_′ = − 48.3 kJ/mol, compared to Δ*G*_0_′ = − 6.9 kJ/mol, based on a redox potential of − 414 mV for 2H^+^/H_2_). Therefore, we will indicate “1 + x” per methylene-THF reduced in the Ech-MetFV complex catalyzed reaction. For the ATP synthase, a H^+^/ATP stoichiometry of 3.6 is assumed (based on a number of 11 c subunits in the *c*-ring of the ATP synthase of *Clostridium paradoxum* (Ferguson et al. 2006; Meier et al. 2006). Then, acetogenesis from H_2_ + CO_2_ is coupled to the synthesis of x/3.6 mol ATP/mol acetate (Fig. 4A), whereas acetogenesis from CO yields 1.1 + (x/3.6) mol ATP/mol acetate (Fig. 4B). This model is also applicable to acetogenesis from other substrates, like glucose (Fig. 5A) or mannitol (Fig. 5B) for *T. kivui*. With glucose, the ATP yield is 0.87 + (x/10.8) ATP/mol acetate. For mannitol, the value is lower with 0.22 + (x/46.8) ATP/mol acetate.

## Conclusion

With the data presented in this report, we have now a much better understanding of the biochemistry and metabolism of different carbon and energy sources in *T. kivui*. We have identified for all but one oxidoreductases the electron carriers. The MetFV-type MTHFR only uses reduced Fd as electron donor, but not NADH or NADPH. Reduced Fd can not be the direct physiological electron donor, but it is hypothesized that the MTHFR is hooked up to the Ech complex. The actual amount of ions translocated is unknown but must be bigger than 1. With H_2_ + CO_2_ as substrate x/3.6 mol ATP/mol acetate are formed, whereas acetogenesis from CO yield 1.1 + (x/3.6) mol ATP/mol acetate. During sugar fermentation, hydrogen is produced by the electron-bifurcating hydrogenase. The NADPH is provided by the Nfn complex. With glucose the ATP yield is 0.87 + (x/10.8) ATP/mol acetate and with mannitol 0.22 + (x/46.8) ATP/mol acetate.

## Supplementary Information

Below is the link to the electronic supplementary material.

Supplementary file1 (DOCX 1098 kb)

## Abbreviations

THF: Tetrahydrofolate (5,6,7,8-tetrahydropteroyl-l-glutamic acid)
BV: Benzyl viologen
MV: Methyl viologen
CFE: Cell-free extract
GA3P: Glyceraldehyde-3-phosphate
GA3P-DH: Glyceraldehyde-3-phosphate dehydrogenase
G6P: Glucose-6-phosphate
G6P-DH: Glucose-6-phosphate deyhdrogenase
Fd: Ferredoxin
PFOR: Pyruvate:ferredoxin oxidoreductase
TPP: Thiamine pyrophosphate
CoA: Coenzyme A
WLP: Wood–Ljungdahl pathway
CODH/ACS: Carbon monoxide dehydrogenase/acetyl-CoA synthase
CooS: Monofunctional CO dehydrogenase
ATP: Adenosine triphosphate
Ech: Energy-converting hydrogenase
Rnf: Rhodobacter nitrogen fixation
HydABC: Electron-bifurcating hydrogenase
MTHFR: Methylene-THF reductase
[4Fe-4S]: Iron-sulfur cluster
[FeFe]: Fe–Fe H-cluster
SDS: Sodium dodecyl sulfate
OD: Optical density
HDCR: Hydrogen-dependent CO_2_ reductase
SEM: Standard error of the mean
FMN: Flavin mononucleotide
FAD: Flavin adenine dinucleotide
TLC: Thin-layer chromatography
