# Supplementary material for: Electron carriers involved in autotrophic and heterotrophic acetogenesis in the thermophilic bacterium Thermoanaerobacter kivui
Source: Extremophiles. 2021 Oct 14;25(5-6):513–26. doi: 10.1007/s00792-021-01247-8 (PMC8578170; doi:10.1007/s00792-021-01247-8)
Supplement: Supplementary file 1 — Supplementary file1 (DOCX 1098 kb) [file 792_2021_1247_MOESM1_ESM.docx]

**Supplementary Information**

for the manuscript:

**Electron carriers involved in autotrophic and heterotrophic acetogenesis in the thermophilic bacterium**

***Thermoanaerobacter kivui***

**Alexander Katsyv^1^, Surbhi Jain^1^, Mirko Basen^2^ and Volker Müller^1^***

^1^Department of Molecular Microbiology & Bioenergetics, Institute of Molecular Biosciences, Johann Wolfgang Goethe University, Frankfurt am Main, Germany

^2^Microbiology, Institute of Biological Sciences, University of Rostock, 18059 Rostock, Germany

^*^correspondence address: Prof. Volker Müller, Department of Molecular Microbiology & Bioenergetics, Institute of Molecular Biosciences, Johann Wolfgang Goethe University, Frankfurt am Main, Germany; Phone: 49-6979829507; Fax: 49-69-79829306;

E-mail: [vmueller@bio.uni-frankfurt.de](mailto:vmueller@bio.uni-frankfurt.de)

**Fig. S1**

**Fig. S2**

**Fig. S3**

**Fig. S4**

**Fig. S5**

**Fig. S6**

**Fig. S7**

**Fig. S8**

**Fig. S9**

**Fig. S10**

**Fig. S11**

**Fig. S12**

**Fig. S13**

**Table S1**


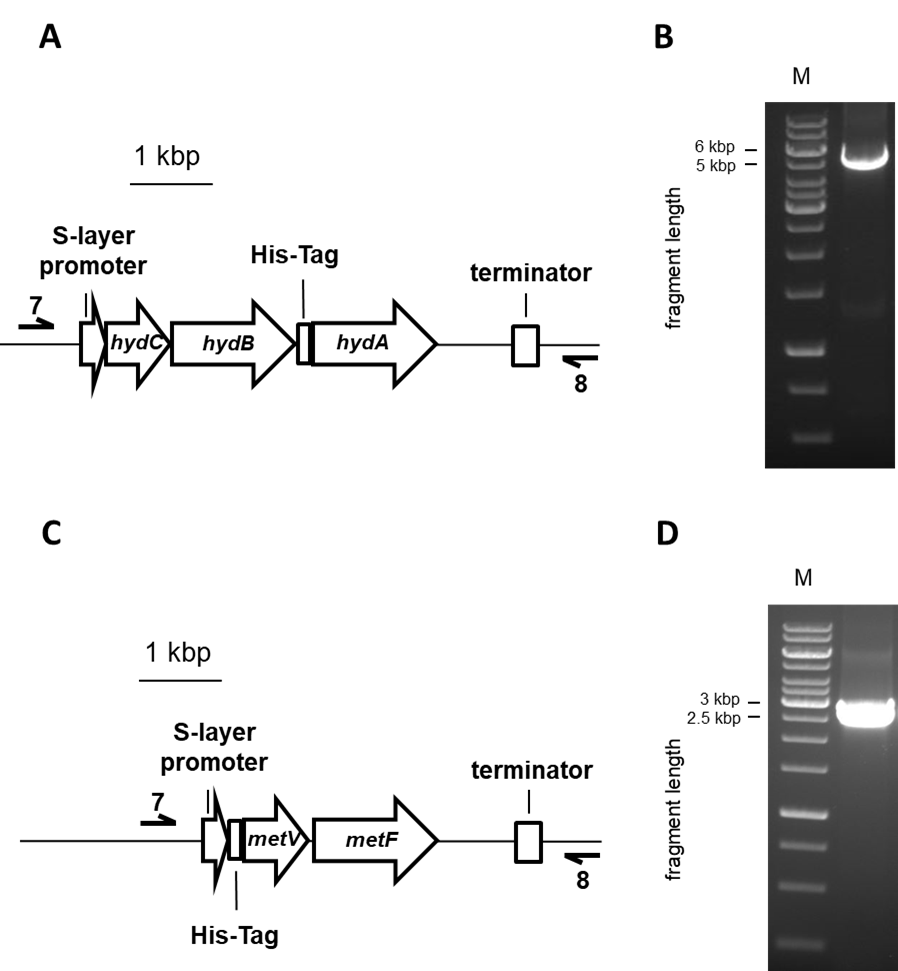


**Fig. S1. Verification of the *pMU131_His-hydABC* and *pMU131_His-metFV* constructs transformed in *T. kivui*.** To verify the nature of the plasmids *pMU131_His-hydABC* and *pMU131_His-metFV* after propagation, *T. kivui* colonies were picked and the plasmids were checked by using primer pairs seq1_for (7)/ seq2_rev (8) (Tab. S1) binding on the *pMU131* backbone and amplifying the complete *His-hydABC* (A) or *His-metFV* (C). The resulting size was 5405 bp (B) or 2768 bp (D). M, Gene Ruler 1 kb DNA ladder.


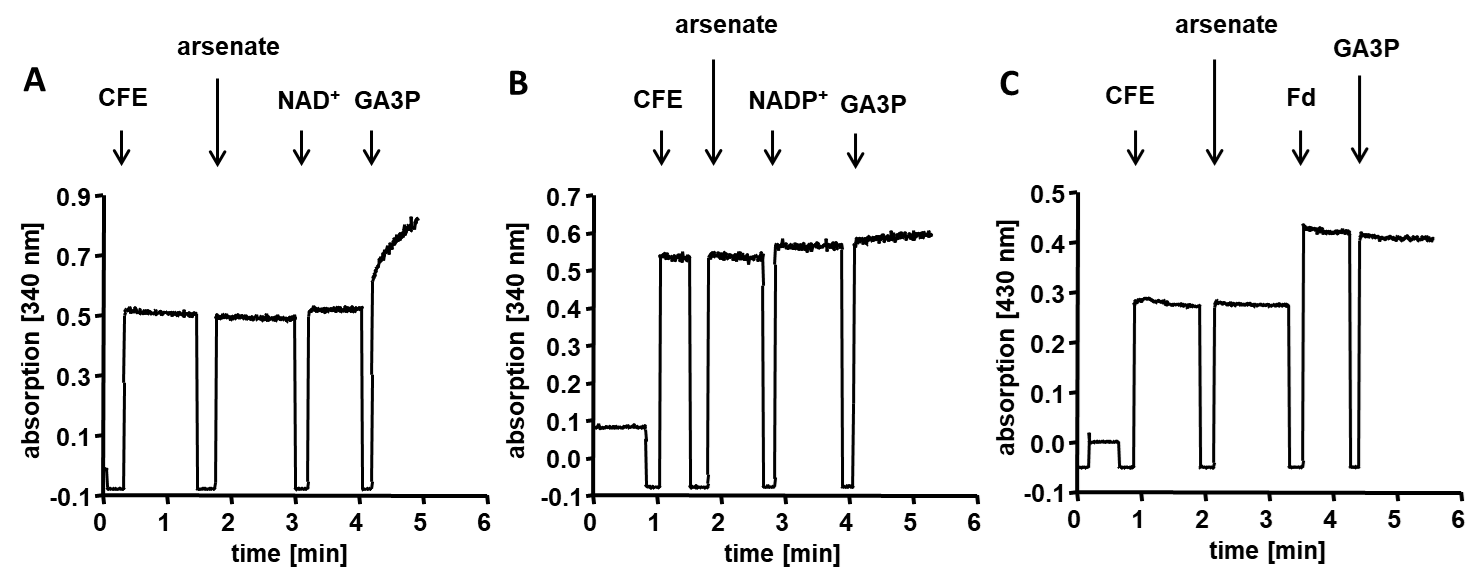


**Fig. S2. NAD^+^-dependent GA3P-DH activity.** GA3P-DH activity was measured in 1.8-ml anoxic cuvettes containing an overall liquid volume of 1 ml under a 100% N_2_ atmosphere (1 × 10^5^ Pa) at 66 °C. The GA3P:NAD^+^/NADP^+^/Fd oxidoreductase assay contained 200 - 500 μg CFE of glucose-grown *T. kivui* cells, 5 mM arsenate and 4 mM NAD^+^ (A) or 4 mM NADP^+^ (B) or 30 μM Fd (C) in buffer C (50 mM Tris/HCl, 10 mM NaCl, 2 mM DTE, pH 7.5). The reaction was started by addition of 1 mM GA3P. Reduction of NAD^+^ or NADP^+^ was monitored at 340 nm and reduction of Fd at 430 nm, respectively.


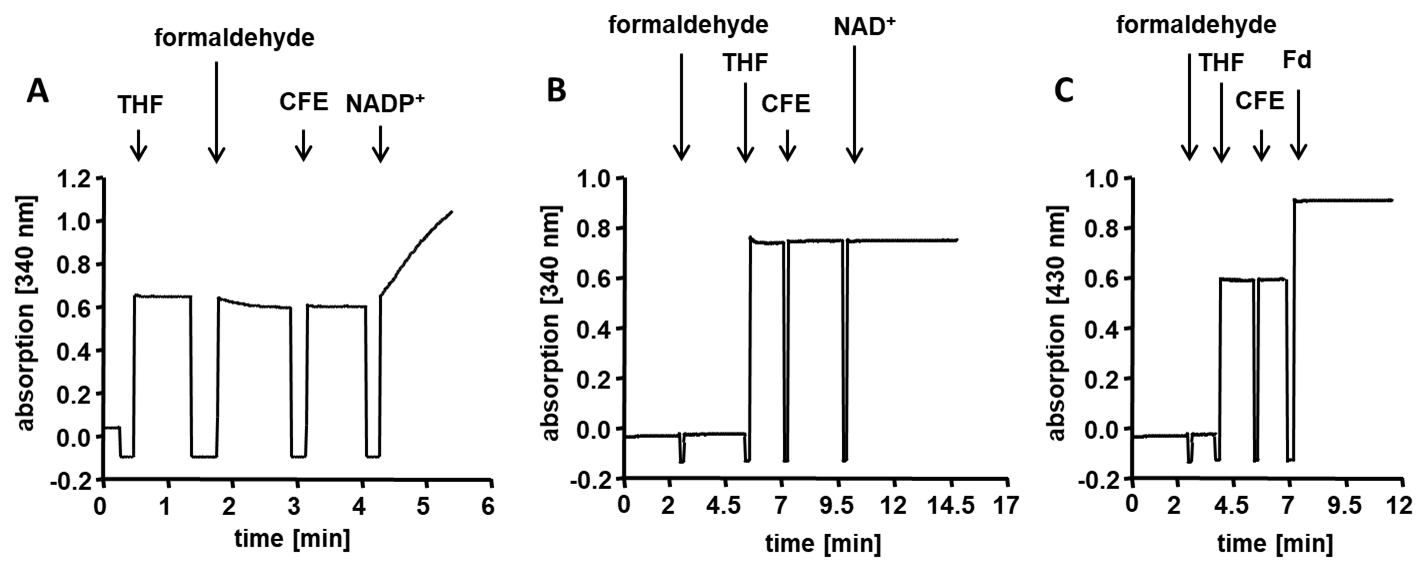


**Fig. S3. NADP^+^-dependent methylene-THF dehydrogenase activity.** Methylene-THF dehydrogenase activity was measured in 1.8-ml anoxic cuvettes containing an overall liquid volume of 1 ml under a 100% N_2_ atmosphere (1 × 10^5^ Pa) at 66 °C. The assay contained 200 - 500 μg CFE of glucose-grown *T. kivui* cells, 1.5 mM formaldehyde and 0.5 mM THF in buffer C (50 mM Tris/HCl, 10 mM NaCl, 2 mM DTE, pH 7.5). The reaction was started by addition of 1 mM NAD^+^ (A), 1 mM NADP^+^ (B) or 30 µM Fd (C). Reduction of NAD^+^, NADP^+^ or Fd was monitored at 340 or 430 nm, respectively.


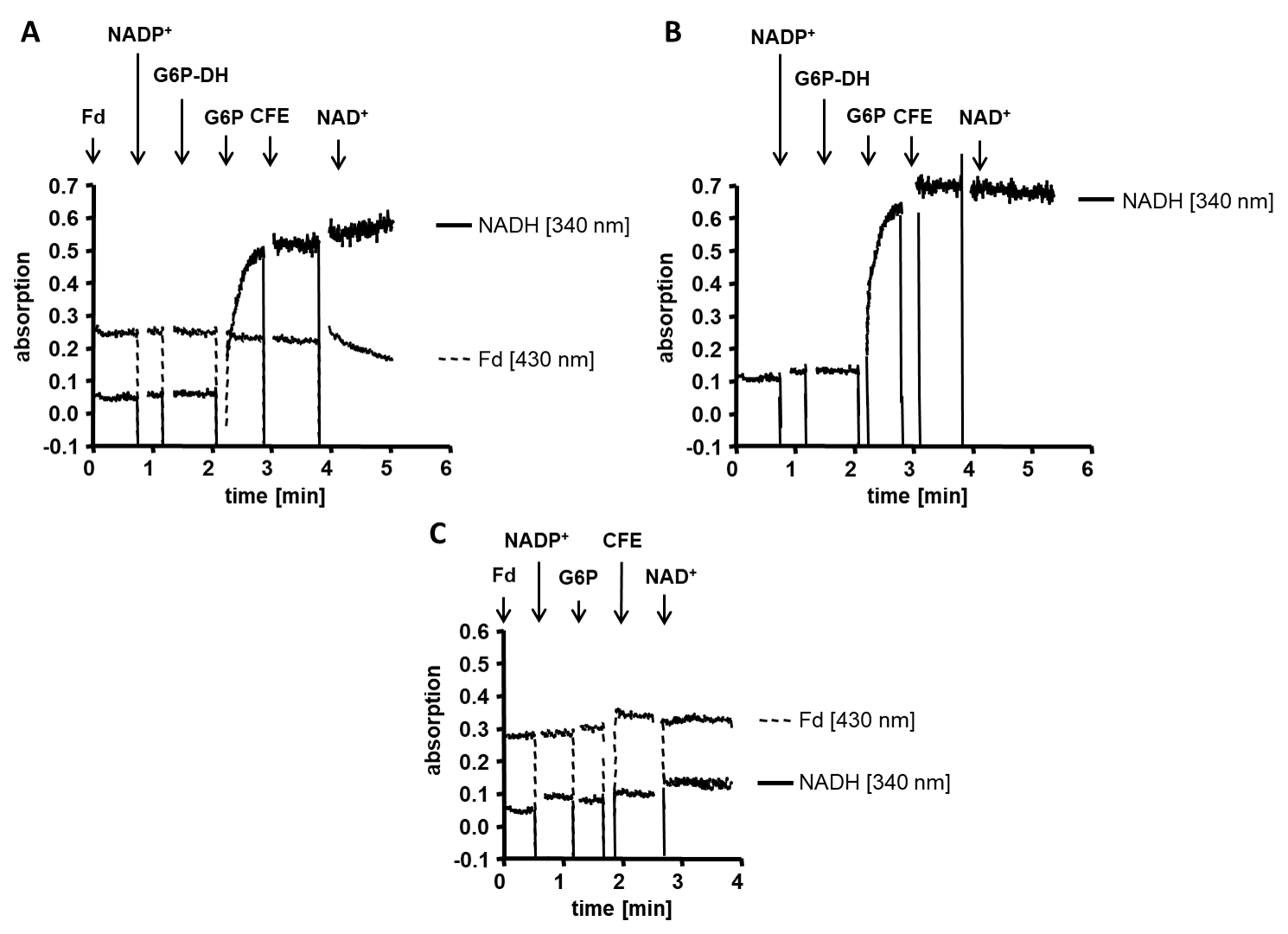


**Fig. S4. NADPH-dependent reduction of NAD^+^ and Fd in CFE of glucose-grown *T. kivui* cells.** NAD^+^-dependent NADPH:Fd ocidoreductase activity was measured in 1.8-ml anoxic cuvettes containing an overall liquid volume of 1 ml under a 100% N_2_ atmosphere (1 × 10^5^ Pa) at 66 °C. The assay contained 410 μg CFE of glucose-grown *T. kivui* cells and 30 µM Fd in buffer C (50 mM Tris/HCl, 10 mM NaCl, 2 mM DTE, pH 7.5). To keep the level of NADPH constant, 0.1 mM NADP^+^ were prereduced with 1 unit G6P-DH and 20 mM G6P (NADP^+^ reducing system) as reported previously ([Kremp et al. 2020](#_ENREF_2)). The reaction was started by addition of 1 mM NAD^+^. In controll assays either Fd (B) or G6P-DH (C) was omitted. Reduction of NAD^+^ and NADP^+^ was monitored at 340 nm and reduction of Fd at 430 nm, respectively.


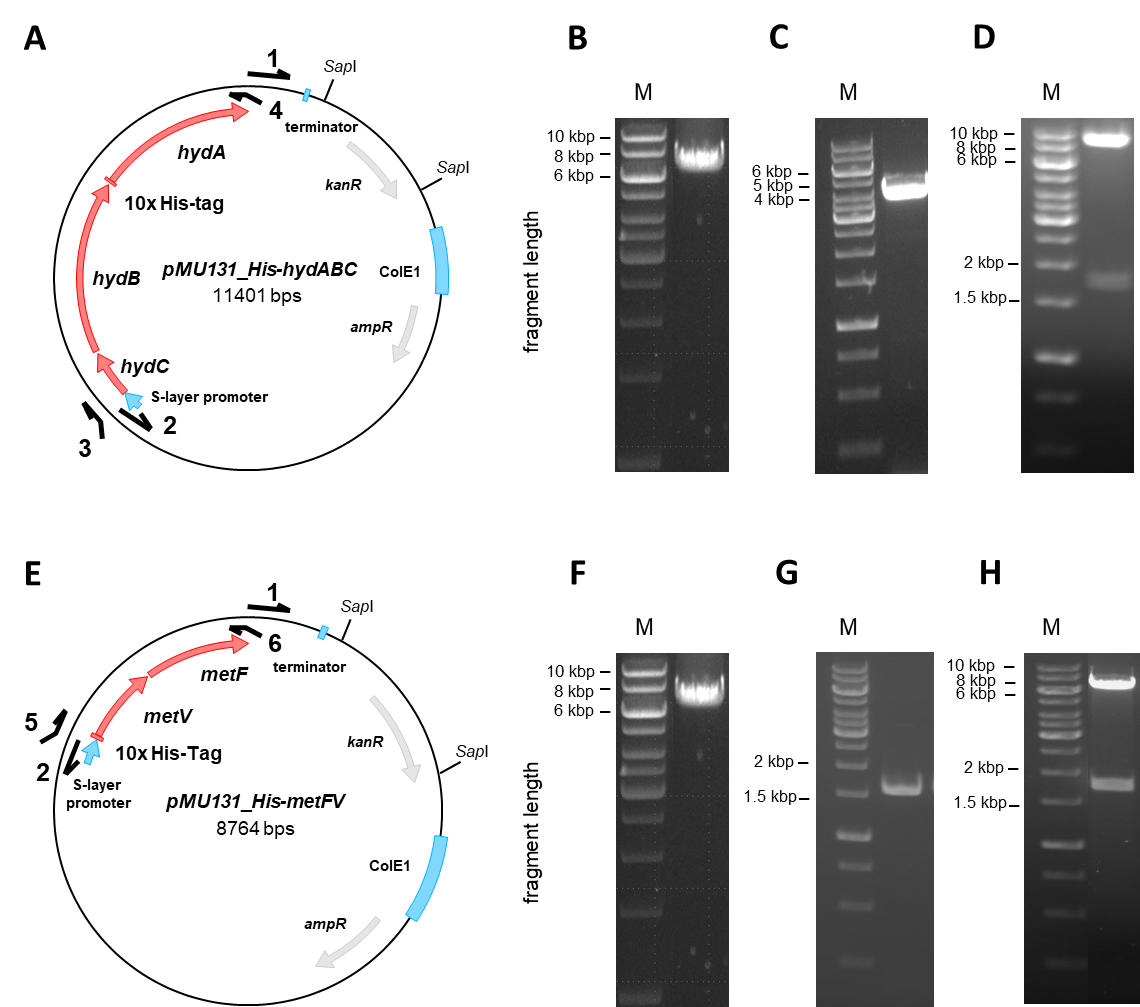


**Fig. S5. Cloning of *pMU131_His-hydABC* and *pMU131_His-metFV*.** For the production of His-HydABC and His-MetFV in *T. kivui* the constructs *pMU131_His-hydABC* and *pMU131_His-metFV* were cloned (A, E). Therefore, *pMU131* backbone, including a S-layer-promoter, was amplified using corresponding primers (1, 2) *via* PCR (B, F) (Tab. S1). *His-metFV* (G) was amplified from genomic DNA of *T. kivui* *via* PCR, using corresponding primers (5, 6) (Tab. S1), containing an additional DNA sequence coding for a 10x His-tag. *HydABC* (C) was amplified from genomic DNA of *T. kivui* *via* PCR using corresponding primers (3, 4) (Tab. S1). Amplified *hydABC* or *His-metFV* and *pMU131* were fused *via* Gibson Assembly and transformed in *E. coli* HB101. In case of *pMU131_His-hydABC*, a DNA sequence coding for a 10x His-tag was introduced at the 3’-end of the gene *hydA* using corresponding primers, *via* site directed mutagenesis. Afterwards, plasmids were isolated and digested with *Sac*I. The resulting sizes for *pMU131_His-hydABC* were 1826 bp and 9575 bp (D) and for *pMU131_His-metFV* 1826 bp and 6938 bp (H). M, Gene Ruler 1 kb DNA ladder.


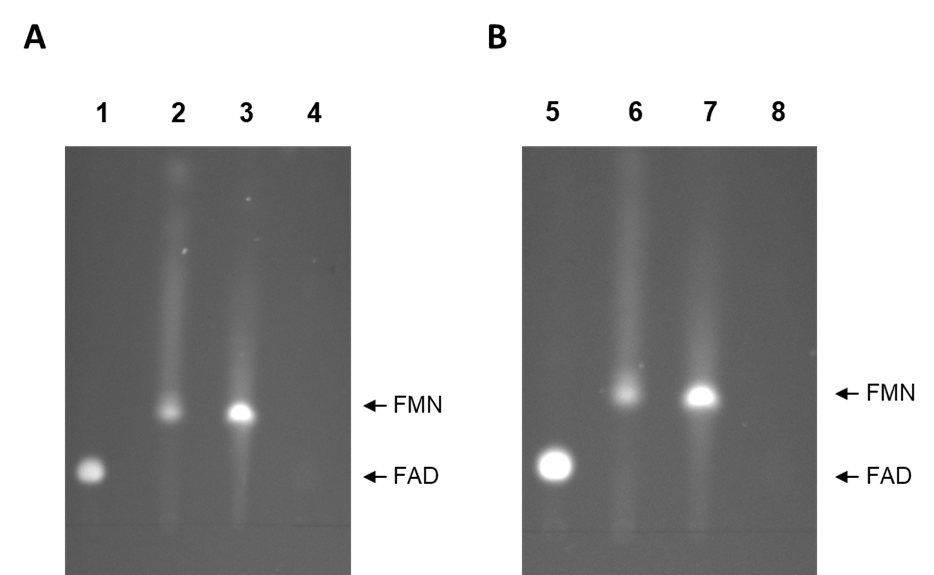


**Fig. S6. Thin layer chromatography (TLC) of the favins extracted from His-HydABC and His-MetFV.** Flavins of ~ 1 nmol His-HydABC (A) or His-MetFV (B) were separated on a TLC plate using 60% [v/v] n-butanol, 15% [v/v] glacial acetic acid and 25% [v/v] H_2_O as the mobile phase. 1 nmol of FAD and FMN was used as standards. For this experiment His-HydABC and His-MetFV was purified with buffers additionally mixed with 10 µM FAD and 10 µM FMN. lane 1 and 5, FAD; lane 2 and 6, FMN; lane 3, His-HydABC; lane 7, His-MetFV; lane 4 and 8, buffer B.


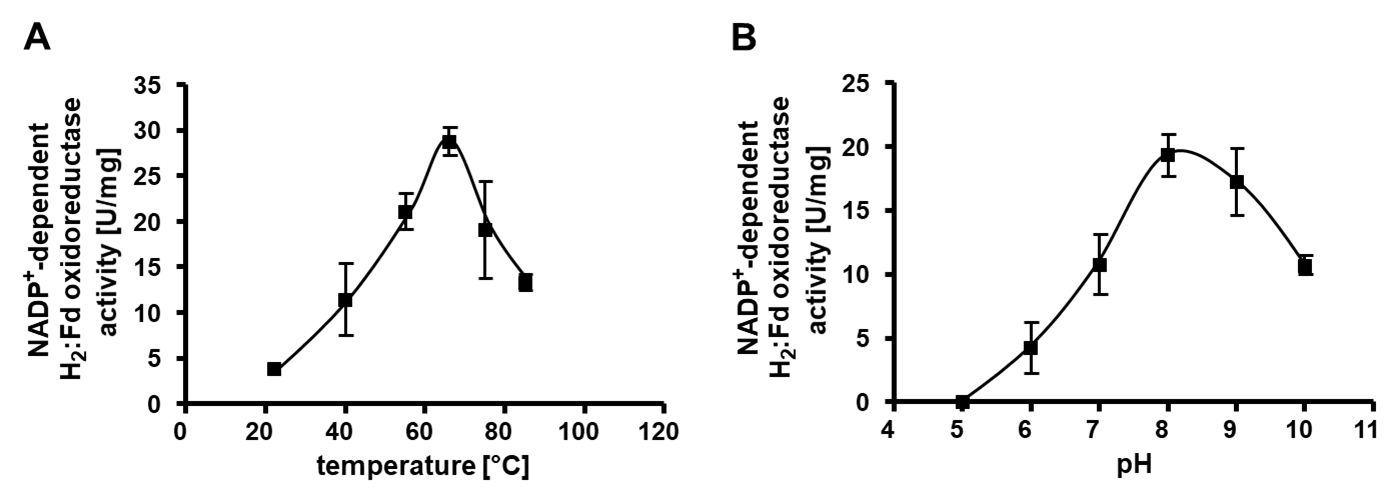


**Fig. S7. pH optimum and temperature profile of purified His-HydABC.** Temperature (A) or pH (B) effect on the NADP^+^-dependent H_2_:Fd oxidoreductase activity was meassured in 1.8-ml anaerobic cuvettes containing an overall liquid volume of 1 ml under a 100% H_2_ atmosphere (2 × 10^5^ Pa) at 22 - 85 °C (A) or 66 °C (B). The assay contained 1 ml of buffer D (50 mM EPPS, 10 mM NaCl, pH 8) (A) or buffer E (50 mM Tris, 50 mM MES, 50 mM CHES, 50 mM CAPS, 50 mM Bis-Tris, 10 mM NaCl, 2 mM DTE, 4 µM Resazurin, pH 5 - 10) (B), 5 - 10 μg His-HydABC, 5 µM FMN and 30 μM Fd. The reaction was started by addition of 4 mM NADP^+^. Reduction of NADP^+^ (340 nm) and Fd (430 nm) were monitored simultaneously. Shown is the average of two measurements from one representative experiment out of two independent replicates. Error bars represent the SEM.


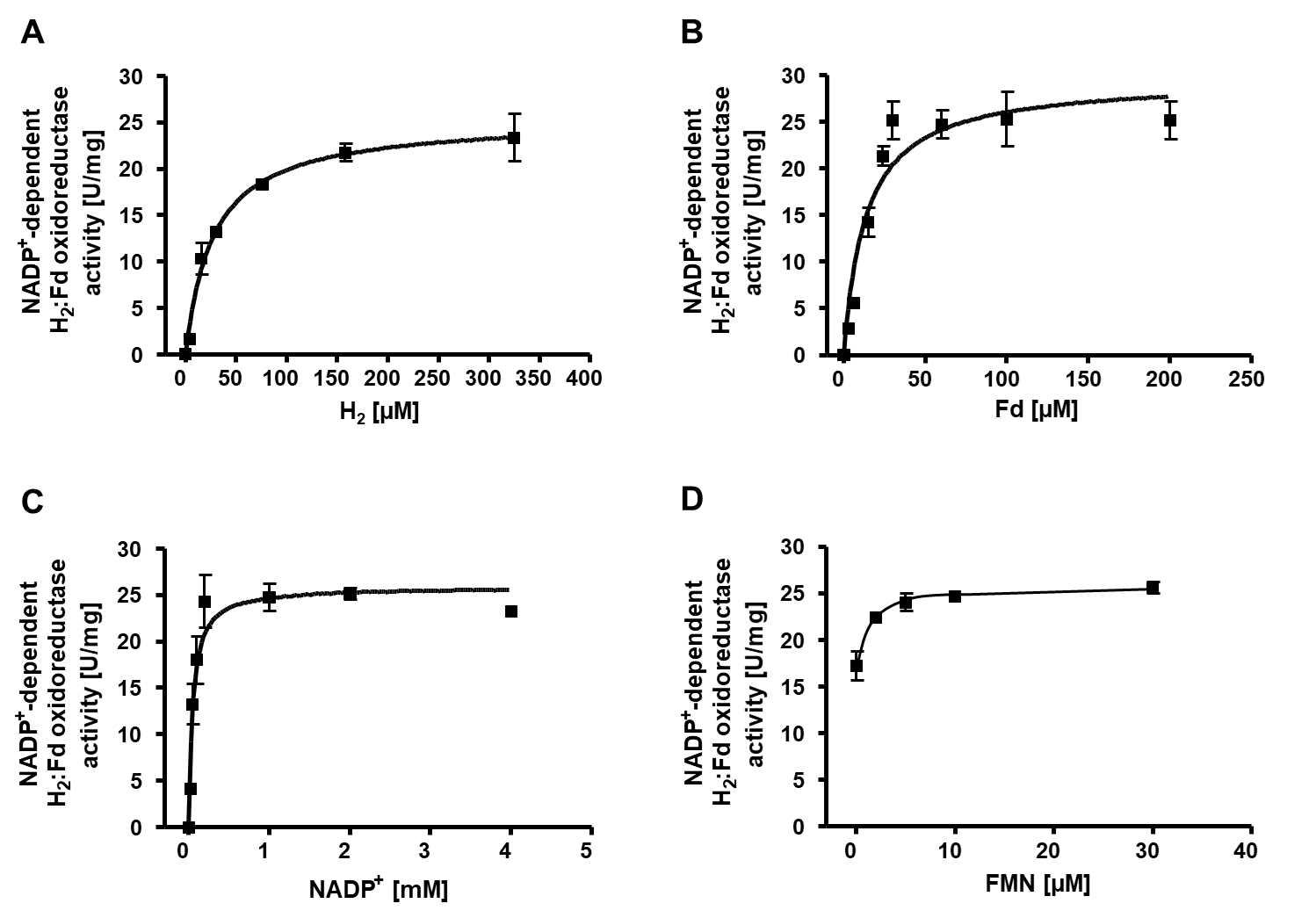


**Fig. S8. H_2_, Fd, NADP^+^ and FMN dependence on His-HydABC activity.** NADP^+^-dependent H_2_:Fd oxidoreductase activity was meassured in 1.8-ml anoxic cuvettes containing an overall liquid volume of 1 ml under a H_2_ atmosphere at 66 °C. The assay contained 1 ml of buffer D (50 mM EPPS, 10 mM NaCl, pH 8), 5 - 10 μg His-HydABC, different amounts of H_2_ (A), Fd (B), NADP^+^ (C) or FMN (D). Reduction of NADP^+^ (340 nm) and Fd (430 nm) were monitored simultaneously. Shown is the average of two measurements from one representative experiment out of two independent replicates. Error bars represent the SEM.


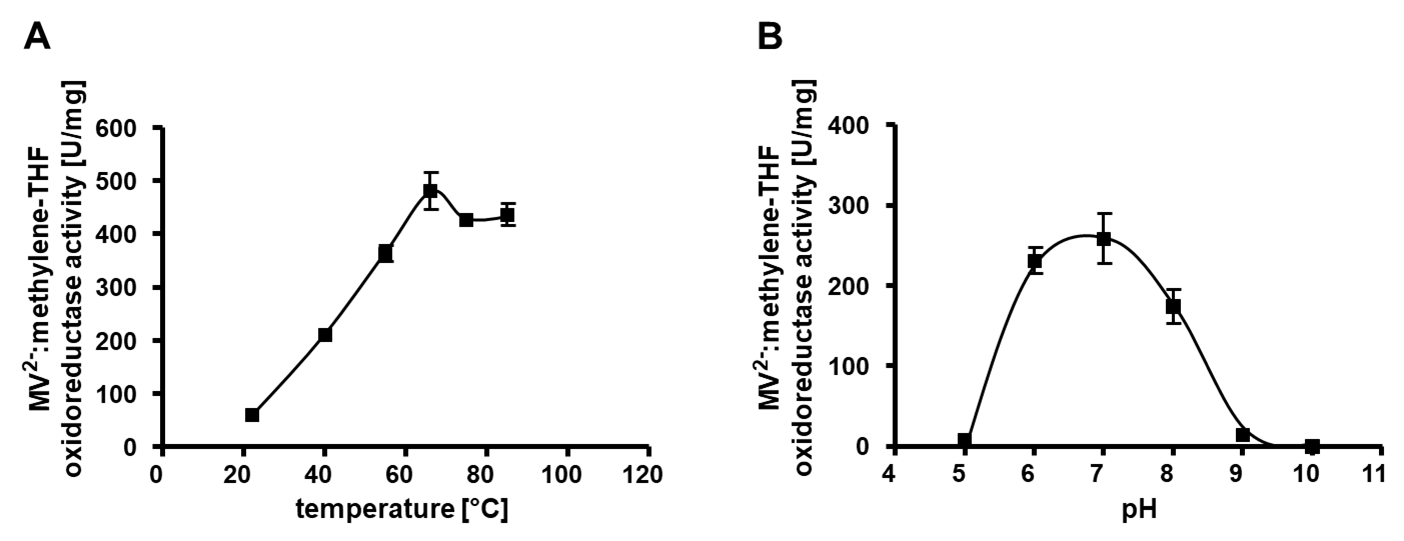


**Fig. S9. pH optimum and temperature profile of purified His-MetFV.** Temperature (A) or pH (B) effect on the MV^2-^:methylene-THF oxidoreductase activity was meassured in 1.8-ml anaerobic cuvettes containing an overall liquid volume of 1 ml under a 100% N_2_ atmosphere (1 × 10^5^ Pa) at 22 - 85 °C (A) or 66 °C (B). The assay contained 1 ml of buffer F (50 mM KP_i_, 20 mM ascorbate, pH 7) (A) or buffer E (50 mM Tris, 50 mM MES, 50 mM CHES, 50 mM CAPS, 50 mM Bis-Tris, 10 mM NaCl, 2 mM DTE, 4 µM Resazurin, pH 5 - 10) (B), 0.5 mM THF, 1.5 mM formaldehyde, 10 mM MV and 5 mM sodium dithionite. The reaction was started by addition of 2 - 5 μg His-MetFV. Oxidation of MV (600 nm) was monitored spectrophotometrically. Shown is the average of two measurements from one representative experiment out of two independent replicates. Error bars represent the SEM.


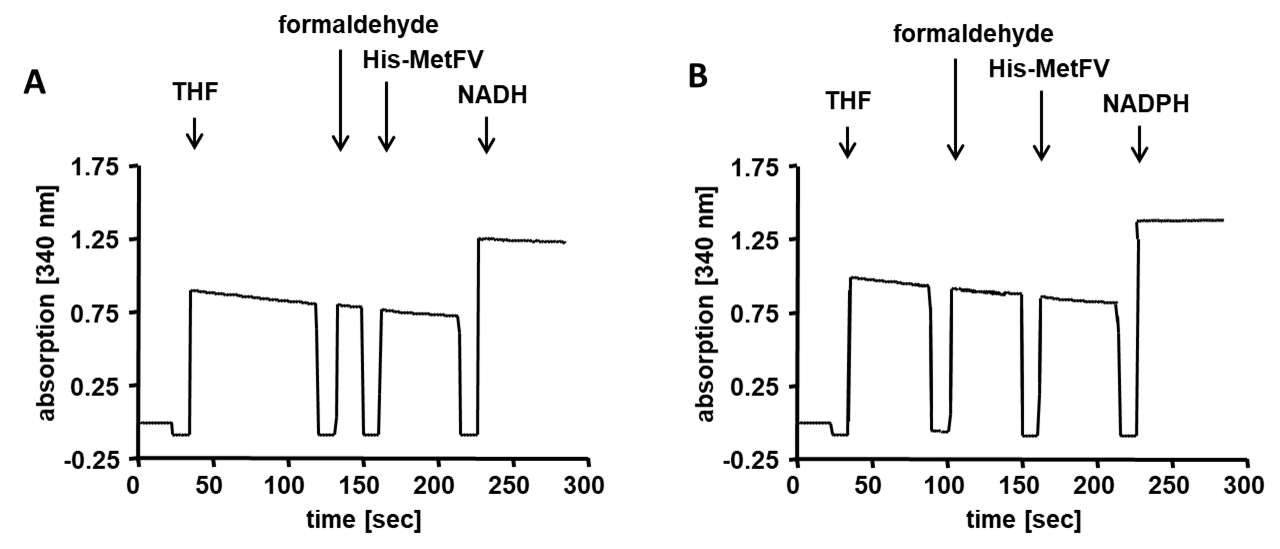


**Fig. S10. NADH/NADPH:methylene-THF oxidoreductase activity of His-MetFV.** MTHFR activity was measured in 1.8-ml anoxic cuvettes containing an overall liquid volume of 1 ml under a 100% N_2_ atmosphere (1 × 10^5^ Pa) at 66 °C. The assay contained 20 μg His-MetFV, 1.5 mM formaldehyde and 0.5 mM THF in buffer F (50 mM KP_i_, 20 mM ascorbate, pH 7). The reaction was started by addition of 0.5 mM NADH (A) or NADPH (B). Oxidation of NADH or NADPH was monitored at 340 nm, respectively.


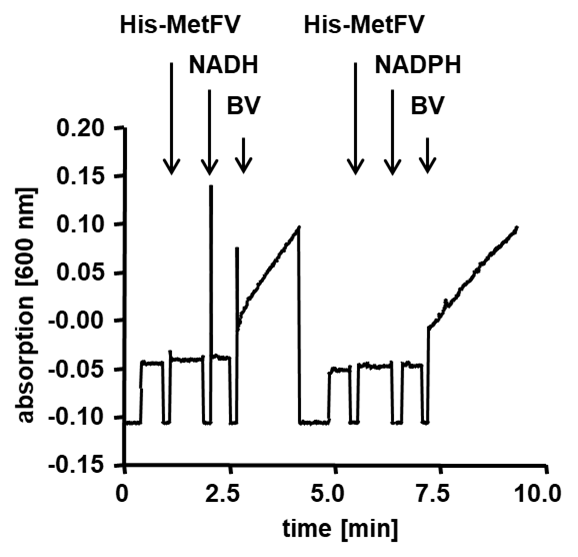


**Fig. S11. NADH/NADPH:BV oxidoreductase activity of His-MetFV.** MTHFR activity was measured in 1.8-ml anoxic cuvettes containing an overall liquid volume of 1 ml under a 100% N_2_ atmosphere (1 × 10^5^ Pa) at 66 °C. The assay contained 30 μg His-MetFV and 0.5 mM NADH or NADPH in buffer F (50 mM KP_i_, 20 mM ascorbate, pH 7). The reaction was started by addition of 10 mM BV. Reduction of BV was monitored at 600 nm, respectively.


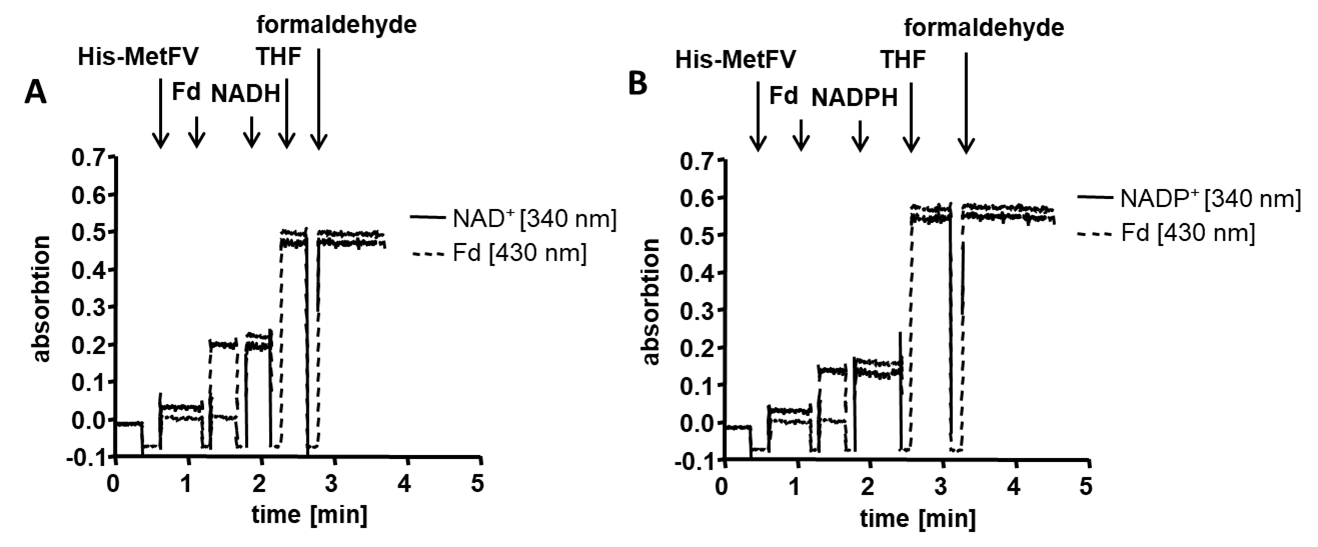


**Fig. S12. Fd-dependent NADH/NADPH:methylene-THF oxidoreductase activity of His-MetFV.** MTHFR activity was measured in 1.8-ml anoxic cuvettes containing an overall liquid volume of 1 ml under a 100% N_2_ atmosphere (1 × 10^5^ Pa) at 66 °C. The assay contained 20 μg His-MetFV, 0.5 mM THF, 0.5 mM NADH (A) or NADPH (B) and 30 μM Fd in buffer F (50 mM KP_i_, 20 mM ascorbate, pH 7). The reaction was started by addition of 1.5 mM formaldehyde. Oxidation of NADH or NADPH and reduction of Fd was monitored at 340 and 430 nm, respectively.


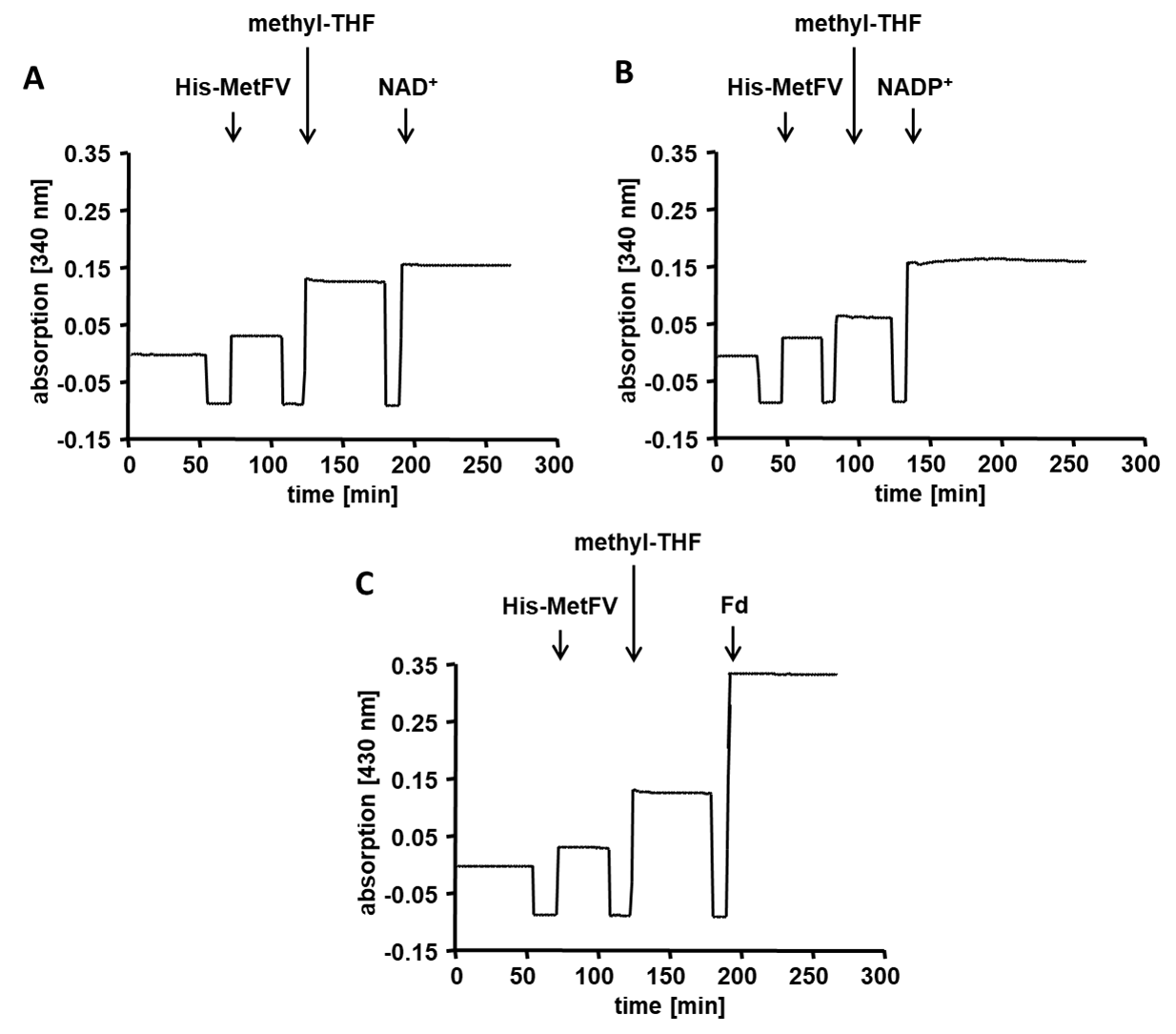


**Fig. S13. Methyl-THF:NAD^+^/NADP^+^/Fd oxidoreductase activity of His-MetFV.** MTHFR activity was measured in 1.8-ml anoxic cuvettes containing an overall liquid volume of 1 ml under a 100% N_2_ atmosphere (1 × 10^5^ Pa) at 66 °C. The assay contained 15 - 30 μg purified His-MetFV and 1 mM methyl-THF in buffer F (50 mM KP_i_, 20 mM ascorbate, pH 7). The reaction was started by addition of 4 mM NAD^+^ (A), 4 mM NADP^+^ (B) or 30 µM Fd (C). Reduction of NAD^+^, NADP^+^ or Fd was monitored at 340 or 430 nm, respectively.

**Tab. S1. Corresponding primers used in this work.**

| **No.** | **Primer** | **Sequence (5’→ 3’)** |
| --- | --- | --- |
| 1 | pMU131_for | TTTTTTAAATTTATCCAGGATAAAAGAGAAGACTC |
| 2 | pMU131_rev | ACAGTCAATCCTCCTCCTTG |
| 3 | HydABCTK_for | caaggaggaggattgactgtATGTGTAATTGCTGCTGC |
| 4 | HydABCTK_rev | tcctggataaatttaaaaaaTTAATATTCTTTCTTTCTTGAGTGATAG |
| 5 | His-MetFVTK_for | caaggaggaggattgactgtATGCACCATCATCATCACCATCATCATCATCATGTCATTACAGAACATAAACCTTTTG |
| 6 | His-MetFVTK_rev | tcctggataaatttaaaaaaTTACAAATTGCACATGTCAAG |
| 7 | seq1_for | TCTAACACAATTATATCATAAGGATTGATA |
| 8 | seq2_rev | AGTATTGTCAATATATTCAAGGCAA |
| 9 | His-HydA_for | catcatcatcatcatAACATGGTAATGTTAACAATAG |
| 10 | His-HydA_rev | gtgatgatgatggtgCATTAAAATCTCACTCCTTTC |

**References**

Kremp F, Roth J, Müller V (2020) The *Sporomusa* type Nfn is a novel type of electron-bifurcating transhydrogenase that links the redox pools in acetogenic bacteria. Sci Rep 10:14872. https://doi.org/10.1038/s41598-020-71038-2

Schönheit P, Wäscher C, Thauer RK (1978) A rapid procedure for the purification of ferredoxin from Clostridia using polyethylenimine. FEBS Lett 89:219-222. https://doi.org/10.1016/0014-5793(78)80221-X
